# Synthesis and optical behaviors of novel triazole fluorescent probes involving solvatochromic behavior, metal ions detection and their antibacterial activity

**DOI:** 10.1038/s41598-026-41364-y

**Published:** 2026-04-07

**Authors:** Hazem M. Elkholy, Wafaa M. Hamada, Marwa N. El-Nahass

**Affiliations:** Chemistry Department, Faculty of Science, Tanta University, Tanta, 31527 Egypt

**Keywords:** Fluorescent probes, Triazole moiety, Solvatochromism, Metal ion detection, Turn-on/turn-off fluorescence, Antibacterial agents, Chemical biology, Chemistry

## Abstract

In this study, two novel fluorescent probes incorporating a triazole moiety, namely (E)-5-((4 H-1,2,4-triazol-3-yl)diazenyl)quinolin-8-ol (**probe 1**) and (E)-3-((4 H-1,2,4-triazol-3-yl)diazenyl)-4-hydroxybenzoic acid (**probe 2**), were synthesized and structurally characterized. Their photophysical properties were evaluated in solvents of varying polarity, showing pronounced solvatochromic shifts and enhanced molecular polarization upon excitation. The chemosensory behavior of the probes toward a series of metal ions, including Na^+^, Mg^2+^, K^+^, Cr^3+^, Fe^3+^, Co^2+^, Ni^2+^, Cu^2+^, Zn^2+^, Cd^2+^, Ba^2+^, and Hg^2+^, was also examined. The probes exhibited high selectivity and sensitivity for Co^2+^ ions via fluorescence turn-on/off mechanisms, with binding constant analysis confirming the strongest affinity for Co^2+^. The limits of detection (LOD) for Co^2+^ were determined to be 0.58 µM for probe (**1**) and 0.06 µM for probe (**2**), both values significantly lower than the maximum permissible limits set by the US Environmental Protection Agency (EPA) and the World Health Organization (WHO) for drinking water. Furthermore, both probes demonstrated promising antibacterial activity against clinically relevant strains, including multidrug-resistant Staphylococcus aureus, Pseudomonas aeruginosa, and Klebsiella pneumoniae, indicating their potential for further development as antimicrobial agents.

**Supplementary Information:**

The online version contains supplementary material available at 10.1038/s41598-026-41364-y.

## Introduction

Azo compounds, characterized by one or more N = N double bonds, represent a versatile class of chemicals with over 2,000 groups synthesized for diverse applications^1,2^. Their unique properties make them valuable in industries such as food, textiles, and pharmaceuticals, as well as in the design of photonic and electro-optical devices^3–7^. The applications of azo compounds are heavily influenced by their molecular structure, the presence of various active groups, and their surrounding environment.

Research has demonstrated that the active groups in azo compounds can promote the formation of different tautomers, with their distribution being significantly affected by molecular structure and environmental factors^3,8–11^. These factors are particularly important in determining both linear and nonlinear optical properties, which play a critical role in the development of advanced optical materials. For nonlinear optical measurements, techniques such as the Z-scan method have gained prominence due to their simplicity and sensitivity^12–15^. Understanding the relationship between azo dye structures, substituents, and their optical responses is key to unlocking new applications in optical and photonic devices.

Beyond their optical applications, azo compounds have attracted increasing attention in chemical sensing owing to their pronounced changes in absorption and fluorescence properties upon interaction with external stimuli. This characteristic is particularly important in environmental monitoring, as rapid industrialization has led to severe contamination of water bodies with toxic heavy metal ions^16–19^. Although certain metal ions are biologically essential, their accumulation beyond permissible limits poses serious risks to ecosystems and human health, necessitating the development of efficient and selective detection strategies. In this regard, chemical sensors based on dye molecules incorporating ion-binding sites provide a promising and cost-effective approach for real-time qualitative and quantitative monitoring of hazardous metal ions in aqueous media^20–23^. Azo-based dyes, in particular, constitute an excellent sensing platform due to their intense coloration, high sensitivity, and selective response toward specific analytes^24–27^. Recently, triazole-containing systems have emerged as powerful building blocks in chemosensor design owing to the strong coordination ability of the triazole ring with metal ions through its nitrogen atoms^28^. Triazole derivatives, accessible through a variety of efficient synthetic routes, have been shown through experimental and spectroscopic investigations to exhibit high binding affinities toward heavy metal ions such as Pb^2+^, Hg^2+^, Cd^2+^, Cu^2+^, and Al^3+^, resulting in distinct fluorescence, redox, and colorimetric responses^29^. Among these systems, triazole Schiff bases have gained particular attention as fluorescent chemosensors due to their structural diversity and favorable photophysical properties^30^. The incorporation of electron-donor and electron-acceptor groups linked via delocalized π-systems promotes efficient intramolecular charge transfer (ICT), leading to pronounced fluorescence modulation upon metal ion coordination. Depending on the metal ion and binding mode, these sensors may operate through ON/OFF, OFF/ON, or ON–OFF–OFF mechanisms, often accompanied by hypsochromic or bathochromic shifts and hypochromic or hyperchromic effects that define their optical signatures^30^. Notably, triazole-based fluorescent probes have demonstrated remarkable performance in the selective detection of Hg^2+^ ions, exhibiting fluorescence quenching or enhancement because of changes in absorption and emission spectra upon coordination. Advances reported between 2010 and 2025 highlight their high selectivity, excellent sensitivity, and low detection limits, underscoring their strong potential for practical applications in environmental monitoring and analytical chemistry, with comparable progress also achieved for Cu^2+^ ion recognition^28,29^.

This study focuses on the synthesis and evaluation of two novel azo dye-based probes: (E)-5-((4 H-1,2,4-triazol-3-yl)diazenyl)quinolin-8-ol (probe 1) and (E)-3-((4 H-1,2,4-triazol-3-yl)diazenyl)-4-hydroxybenzoic acid (probe 2). These probes were designed to function as nonlinear optical materials and sensors for metal ion detection in aqueous systems. These fluorescent probes demonstrated the ability to detect various metal ions like Na^+^, Mg^2+^, K^+^, Cr^3+^, Fe^3+^, Co^2+^, Ni^2+^, Cu^2+^, Zn^2+^, Cd^2+^, Ba^2+^, and Hg^2+^ with distinct color changes visible to the naked eye, depending on the solvent system. The calculated limits of detection for both probes highlight their practical utility and high sensitivity, making them promising candidates for environmental monitoring and industrial applications. This work bridges the gap between the structural and optical properties of azo dyes and their environmental applications, offering insights into their potential as nonlinear optical materials as well as sensors for metal ions in water.

## Experimental

### Materials

Chemical reagents and solvents were procured from commercial suppliers and used without further purification. 4 H-1,2,4-triazol-3-amine, quinolin-8-ol and 4-hydroxybenzoic acid were sourced from Sigma-Aldrich Chemical Co. (St. Louis, MO, USA). The investigated metal salts included, chromium chloride hexahydrate (CrCl_3_·6H_2_O), ferric chloride (FeCl_3_), cobalt chloride hexahydrate (CoCl_2_·6H_2_O), nickel chloride hexahydrate (NiCl_2_·6H_2_O), copper chloride dihydrate (CuCl_2_·2H_2_O), zinc chloride (ZnCl_2_), Barium chloride dihydrate (BaCl_2_·2H_2_O) and mercury chloride (HgCl_2_).

Solvents used were methanol (MeOH), ethanol (EtOH), propanol (PrOH, acetonitrile (ACN), dimethyl sulfoxide (DMSO), chloroform (CHCl_3_), dimethylformamide (DMF), and heptane.

### Synthesis of (E)-5-((4 H-1,2,4-triazol-3-yl)diazenyl)quinolin-8-ol (probe 1) and (E)-3-((4 H-1,2,4-triazol-3-yl)diazenyl)-4-hydroxybenzoic acid (probe 2)

**Fig. 1:**
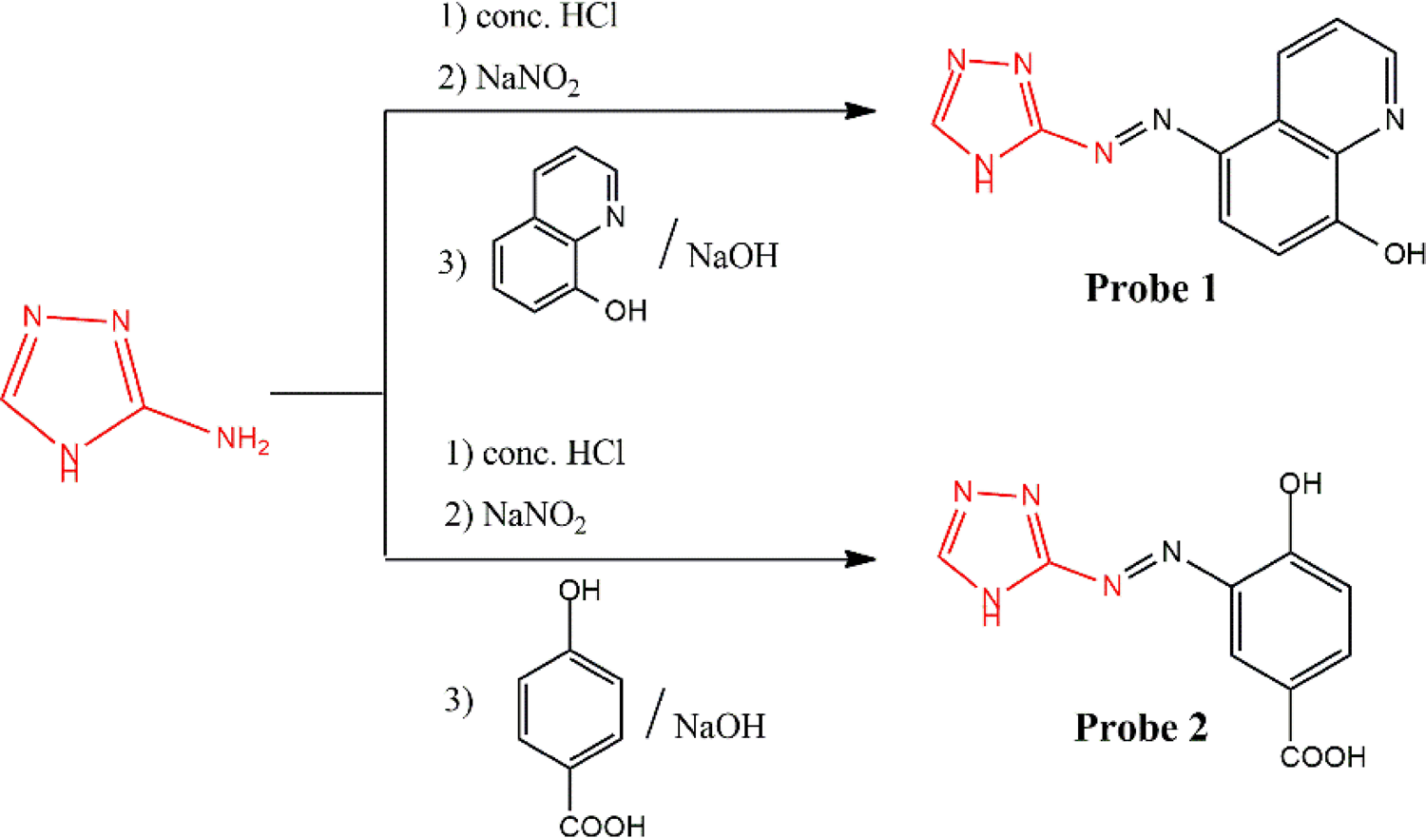
Synthetic pathway of fluorescent probes **(1)** and **(2)**.

4 H-1,2,4-triazol-3-amine (1 g, 11.89 mmol) was dissolved in concentrated HCl (8.5 N, 2 mL) and distilled water (40 mL), then cooled in an ice bath at 0 °C. A cooled solution of sodium nitrite (0.83 g, 11.89 mmol) in distilled water (10 mL) was added dropwise to amine solution to generate the diazonium salt solution. Subsequently, a solution of quinolin-8-ol (1.73 g, 11.89 mmol) and/or 4-hydroxybenzoic acid (1.64 g, 11.89 mmol) dissolved in NaOH (2.5 g, 62.5 mmol) and distilled water (40 mL) was added to the diazonium salt solution with continuous stirring for 60 min. The pH of the reaction mixture was adjusted to 6.5, and the resulting precipitate was filtered, dried and purified using silica gel column chromatography with methanol as the eluent, Fig. 1.

**(E)-5-((4 H-1**,**2**,**4-triazol-3-yl)diazenyl)quinolin-8-ol (probe 1)**: Yield = 65%,m.*p* = 250 °C (decomp.). FT-IR: *v* (cm^− 1^) 3417 (st. O-H), 3255 (st. N-H), 3095 (ar. CH), 1565 (C = N), 1470 (N = N), 1323 (C-N). ^1^H-NMR (DMSO-*d*_*6*_): δ (ppm) = 6.77–9.71 (*m*, 5 H, CH of quinoline ring), 8.85 (*s*, 1H, CH of 1,2,4-triazol ring), 9.36 (*s*, 1H, NH of 1,2,4-triazol ring). ^13^C-NMR (DMSO-*d*_*6*_): δ (ppm) = 111.53 (C5), 121.97 (C4, C10), 123.61 (C8), 127.62 (C9), 129.05 (C3, C7), 136.13 (C1, C11), 142.85 (C2), 148.28 (C6). MS: Calculated M = 240.20 (C_11_H_8_N_6_O); Found: M = 240.06 (16.84%). M.A. (%);Calcd: C (55.00), H (3.36), N (34.98). Found: C (55.11), H (3.39), N (34.77).

**(E)-3-((4 H-1**,**2**,**4-triazol-3-yl)diazenyl)-4-hydroxybenzoic acid (probe 2)**: Yield = 82%,m.*p* = 225–227 °C. FT-IR: *v* (cm^− 1^) 3428 (st. O-H), 3130 (st. N-H), 3050 (ar. CH), 1633 (C = O), 1595 (C = N), 1474 (N = N), 1280 (C-N). ^1^H-NMR (DMSO-*d*_*6*_): δ (ppm) = 6.95 (*d*, 2 H, ar. CH of benzene ring), 7.84 (*d*, 2 H, CH of benzene ring and NH of 1,2,4-triazol ring), 8.57 (*s*, 1H, CH of 1,2,4-triazol ring). ^13^C-NMR (DMSO-*d*_*6*_): δ (ppm) = 116.71 (C6, C7), 125.86 (C4, C5), 145.75 (C3), 146.73 (C1), 162.83 (C2, C8), 168 (C9). MS: Calculated M = 233.20 (C_9_H_7_N_5_O_3_); Found: M = 233.55 (38.09%). M.A. (%);Calcd: C (46.36), H (3.03), N (30.03). Found: C (46.43), H (3.07), N (29.95).

### Characterization

The Fourier-transform infrared (FT-IR) spectrum was acquired using a JASCO FT/IR-4100 spectrophotometer with KBr pellets in the 4000 to 400 cm^− 1^ range, and the processed spectrum utilized the KBr disc technique. A Perkin-Elmer 240 CHN elements analyzer was used to carry out the elemental analysis. The NMR spectra were registered employing a Bruker AC spectrometer operating at 400 MHz. Mass spectra were produced using a Finnigan MAT 8222 EX mass spectrometer with electron energy of 70 eV. The C, H, and N contents of the two probes were determined in Micro Analytical Center, Cairo University. Electronic absorption spectra were obtained using an Agilent Cary Eclipse UV–Vis scanning spectrophotometer, whereas fluorescence emission spectra were recorded with an Agilent Cary Eclipse spectrofluorometer at excitation wavelengths of 450 nm and 325 nm for probes (1) and (2), respectively. Electronic absorption spectra were measured using an Agilent Cary Eclipse UV-Vis scanning spectrophotometer, while emission spectra were recorded with an Agilent Cary Eclipse fluorescence spectrophotometer.

### Spectral measurements

#### Effect of solvents

To examine the effect of solvents, a precise volume of stock solutions (approximately 10^˗^³ M) was diluted to the desired concentration in calibrated measuring flasks. For all solvents, 8 × 10^˗5^ and 3 × 10^˗5^ M solutions of the fluorescent probes (1) and (2) were utilized, respectively. The stock solution, initially prepared in ethanol (EtOH), was diluted by adding 0.4 and 0.15 mL of this solution to a 5 mL volumetric flask containing the target solvent (MeOH, EtOH, PrOH, ACN, DMSO, CHCl_3_, DMF, or heptane).

#### Metal ions detection

Stock solutions of the investigated fluorescent probes (1) and (2) were prepared in EtOH at a concentration of 1 × 10⁻³ M. Similarly, solutions of metal chloride salts, including Na^+^, Mg^2+^, K^+^, Cr^3+^, Fe^3+^, Co^2+^, Ni^2+^, Cu^2+^, Zn^2+^, Cd^2+^, Ba^2+^, and Hg^2+^, were prepared at concentration (1 × 10⁻^2^ M). During the titration process, the concentration of the fluorescent probes (1) and (2) was kept constant at 8 × 10^˗5^ and 3 × 10^˗5^ M, while the concentration of metal ions was systematically varied from 0 to 8.0 × 10⁻^3^ M. Steady-state absorption and emission techniques were utilized, with a 10 min waiting period after mixing the solutions to ensure uniformity and equilibrium before starting each experimental cycle.

### Antibacterial activity

The antibacterial activity of probes (1) and (2) (0.02 g dissolved in 1 mL DMSO) were evaluated against selected pathogenic microorganisms using the agar well diffusion method in triplicate. The tested pathogens included Gram-negative bacteria (*Pseudomonas aeruginosa* and *Klebsiella pneumoniae*) and Gram-positive bacteria (*Staphylococcus aureus*). Prior to testing, the bacterial strains were precultured overnight on nutrient agar plates. These precultured bacteria were then swabbed onto separate nutrient agar plates, where 0.8 mm wells were created using a sterilized cork borer. Each well was filled with 100 µL of either the sample suspension or the negative control (sterilized distilled water). The plates were incubated at 37 °C for 24 h, after which the inhibition zones were measured. Dimethyl sulfoxide (DMSO) served as the negative control^31,32^.

## Results and discussion

### Structural characterization of the investigated fluorescent probes

The preparation of two azo dye-based probes (1) and (2) is outlined in Fig. 1 and their chemical structures were elucidated using different spectroscopic techniques. Initially, 4 H-1,2,4-triazol-3-amine was diazotized using sodium nitrite, producing 4 H-1,2,4-triazole-3-diazonium chloride. This diazonium salt was then coupled with quinolin-8-ol and/or 4-hydroxybenzoic acid, resulting in probes (1) and (2), respectively. FT-IR spectrum (Fig. S1) of probe (1) provided characteristic stretching bands for O-H, N-H and azo group (-N = N-) at 3417, 3255 and 1470 cm^− 1^, respectively. Its^1^ H-NMR chart (Fig. S2A) displayed multiplet signals corresponding to five aromatic hydrogens of quinoline ring in the range 6.77–9.71 ppm, along with singlet peaks for CH and NH of triazol ring at 8.85 and 9.36 ppm. ^13^C-NMR spectrum (Fig. S3A) provided different types of carbon, especially the carbon (C6) attached to the hydroxyl group at 148.28 ppm. Moreover, the carbon (C2) of triazole ring linked to the azo group appeared at 142.85 ppm and the other carbon of triazole and quinoline rings (C1, C11) attached to nitrogen atoms displayed at136.13 ppm.

Similarly, FT-IR spectrum (Fig. S1) of probe (2) illustrated the stretching bands for O-H, carbonyl (C = O) and azo group (-N = N-) at 3428, 1633 and 1474 cm^− 1^, respectively. The^1^ H-NMR spectrum (Fig. S2B) showed a doublet peak at 6.95 ppm corresponding to two aromatic benzene protons and another doublet at 7.84 ppm attributed to two benzene protons along with the NH proton. A singlet peak at 8.57 ppm was assigned to the CH proton of the 1,2,4-triazole ring. Additionally, its^13^ C-NMR spectrum (Fig. S3B) showed the carboxylic group carbon (C9) at 168 ppm, the C-OH (C8) carbon and carbon of triazole ring attached to the azo group (C2) at 162.83 ppm in addition to the other carbon of triazole ring (C1) and the carbon of benzene ring attached to azo group (C3) at 146.73 and 145.75 ppm, respectively.

The mass spectrum of probe (**1**) showed various mass fragmentations as detailed in Fig. S4. Its molecular ion peak (M^+^) with a formula C_11_H_8_N_6_O, appeared at *m/z* = 240.06 with a relative abundance of 16.84%. The base peak, corresponding to the fragment C_2_H_2_N_3_, was observed at *m/z* = 67.71 with a relative abundance of 100%. The fragmentation process begins with the loss of a hydroxyl group (-OH), resulting in the fragment C_11_H_7_N_6_ (*m/z* = 223.22, 27.93%). This fragment subsequently eliminates an NH group, forming C_11_H_8_N_5_ (m/z = 210.31, 41.62%) which then undergoes cleavage of carbon atom and amino group to produce the moiety C_10_H_7_N_4_ (*m/z* = 183.49, 3.42%). An alternative pathway involves the loss of hydroxy quinoline moiety, yielding the azo-triazole moiety C_2_H_3_N_5_ moiety (*m/z* = 97.11, 43.95%). This moiety further releases various groups, such as three NH units and a carbon atom, ultimately forming the CH_4_N_2_ fragment with *m/z* = 45.12 (70.49%).

Furthermore, Fig. S5 presented the different fragmentation signals and proposed fragmentation pathway of probe (2). The base peak, corresponding to C_7_H_6_N_4_O, was observed at *m/z* = 162.71 with a relative abundance of 100%, while the molecular ion peak (M⁺) for C_9_H_7_N_5_O_3_ appeared at *m/z* = 233.55 with relative abundance of 38.09%. Probe (2) loses two hydrogens, forming C_9_H_4_N_5_O_3_ (*m/z* = 229.69, 38.31%) followed by release hydroxyl group to produce C_9_H_4_N_5_O (*m/z* = 214.22, 57.53%). Subsequently, the loss of a cyano group results in the fragment C_7_H_4_N_2_O_2_ (*m/z* = 148.01, 44.88%). Additionally, the compound loses another cyano group, yielding the fragment C_8_H_6_N_4_O_3_ moiety at *m/z* = 205.00 (54.38%) which then undergoes further nitrogen atom loss to form C_7_H_5_O_3_ at *m/z* = 137.26 (24.26%). Finally, the peak corresponding to the triazole ring (C_2_H_3_N_3_) is clearly observed at *m/z* = 69.86 with a relative abundance of 20.18%.

### Solvent effects on the electronic spectra of the investigated fluorescent probes (1) and (2)

**Fig. 2:**
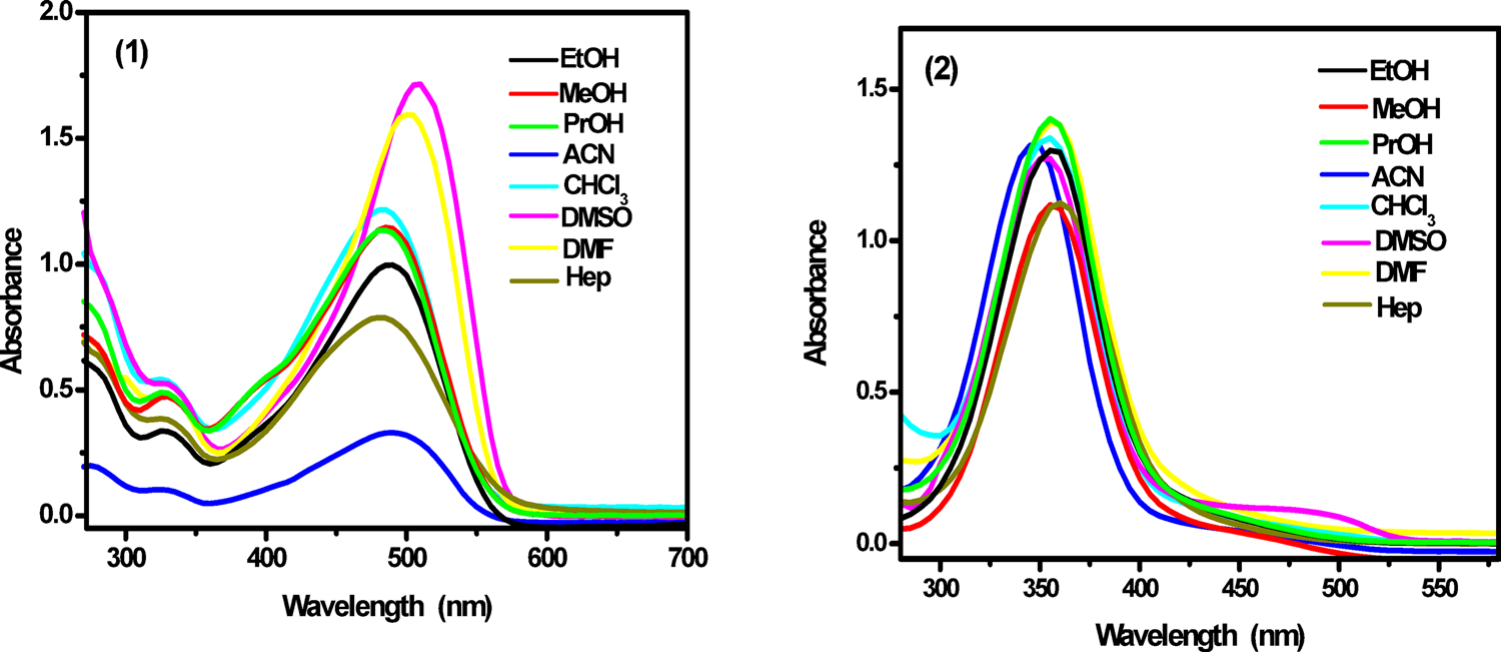
Absorption spectra of the investigated fluorescent probes **(1)** and **(2)** in different solvents of various polarities.

The electronic spectra of fluorescent probes (1) and (2) were examined in various solvents at a concentration of approximately 8 × 10^˗5^ and 3 × 10^˗5^M. The solvents used included: (i) polar protic solvents such as MeOH, EtOH, and PrOH; (ii) polar aprotic solvents such as ACN, DMF, and DMSO; and (iii) nonpolar solvents such as Hep and CHCl_3_. Figure 2 displays the electronic absorption spectra of probes (1) and (2) in the three solvent types: polar protic, polar aprotic, and nonpolar, while Table 1 presents the λ_max_ values across these solvent categories. The characteristic spectral band (λ_max_) appears within the range of 347–509 nm with extinction coefficient ranges from 4000 to 46,666 Lmol^− 1^ cm^− 1^, depending on both the solvent’s properties and the probe’s structure. The data in Table 1 indicate that the absorption maxima of fluorescent probes (1) and (2) vary with solvent type, with maximum shifts of Δλ = 30 nm and 14 nm, respectively. These shifts in spectral position serve as a tool to analyze the interactions between the solute and solvent.

**Fig. 3:**
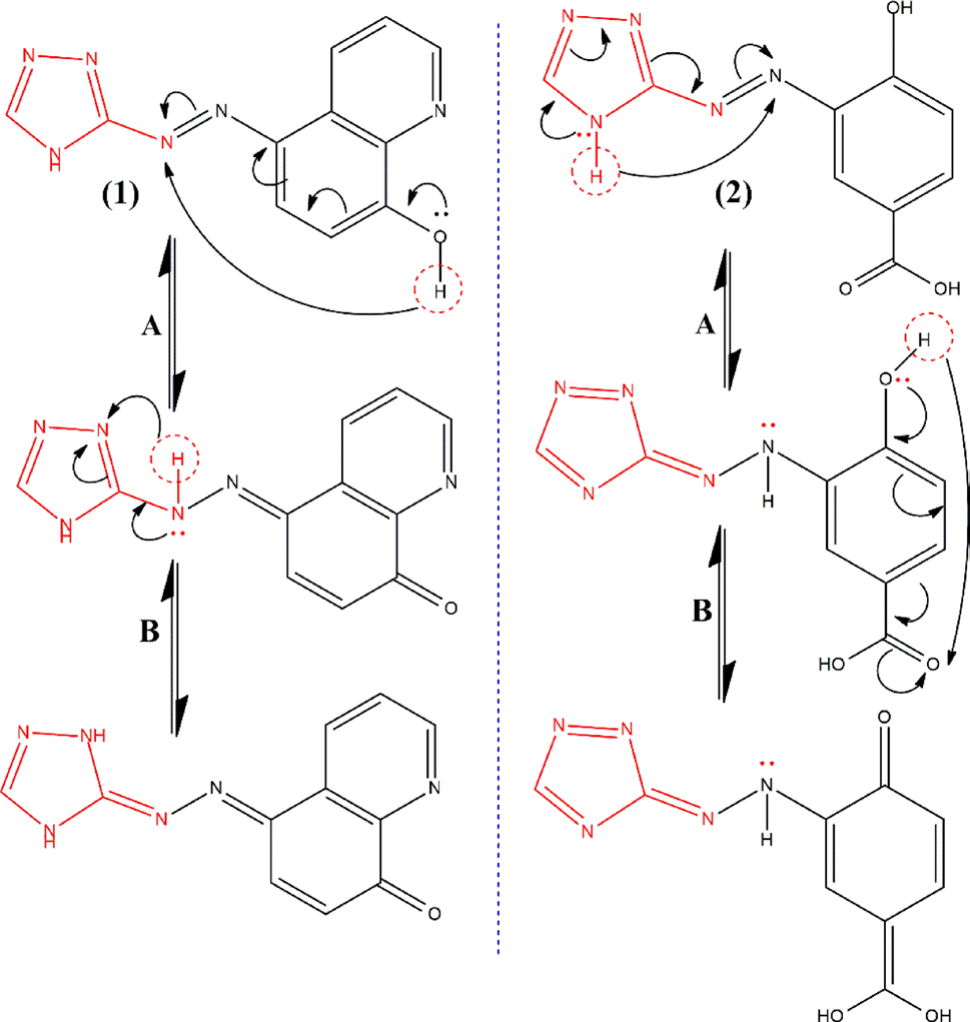
Resonance structures illustrating the donor and acceptor moieties of the investigated dyes.

The broadness of the electronic spectra suggests a significant charge transfer (CT) character, predominantly originating from 8-hydroxy quinoline ring to the triazole moiety in probe (1) and from triazole moiety to 4-hydroxy carboxylic fragment in probe (2) via the azo linkage, Fig. 3. For probe (1), the absorption band exhibits a bathochromic shift from the nonpolar solvent (Hep; λ_max_ = 479 nm) to the polar solvent (DMSO; λ_max_ = 509 nm). This solvatochromism arises from the differential solvation of the ground and Franck-Condon excited states due to the absorption of visible electromagnetic radiation. As the solvent polarity increases, the excited state experiences greater stabilization through solvation compared to the ground state. Moreover, probe (1) shows a more pronounced red shift in DMF and DMSO than in other solvents. This behavior is attributed to the high basicity and hydrogen-bond-accepting capabilities of DMF and DMSO. These solvents stabilize the excited state of probe (1) more than the ground state, leading to a bathochromic shift. It is proposed that intermolecular hydrogen bonding with DMF and DMSO forms a solvated complex, further stabilizing the excited state^33^.

The situation is some extent different for probe (2) as the more polar solvents induce a blue shift of the absorption band. It exhibits a hypsochromic shift from the nonpolar solvent, Hep (λ_max_ = 361 nm) to the polar solvent, DMSO (λ_max_ = 354 nm). The hypsochromic shift (due to the presence of auxochrmoes OH and COOH) with increasing solvent polarity arises due to solute-solvent interactions, particularly hydrogen bonding, which stabilizes the ground state more than the excited state. This differential stabilization increases the energy gap, leading to absorption at shorter wavelengths. In nonpolar solvents, intramolecular hydrogen bonding lowers the energy difference between molecular orbitals, while in polar solvents, intermolecular hydrogen bonding with the solvent disrupts this effect, further stabilizing the ground state. The shift is more pronounced in n-π* transitions due to stronger interactions of lone electron pairs with the solvent, whereas π-π* transitions exhibit a smaller shift due to reduced electron delocalization^34^, Fig. 3.

**Fig. 4:**
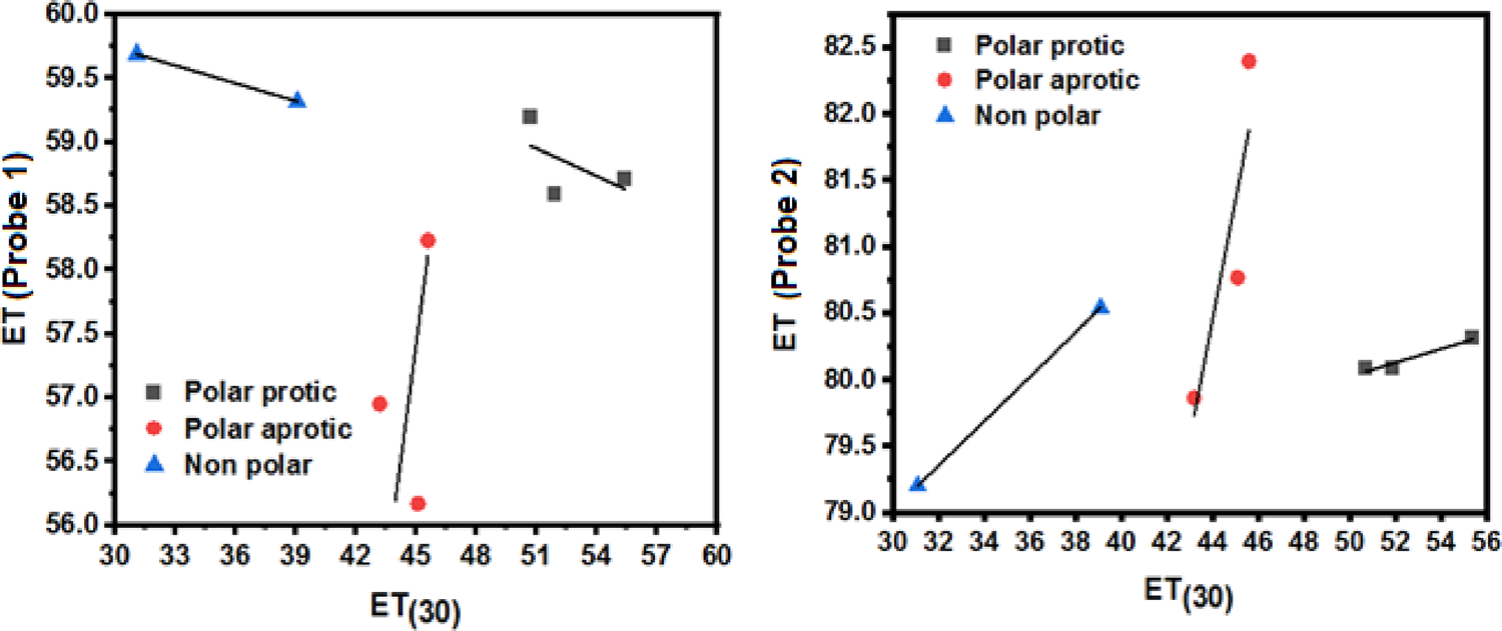
Plot of ET (probes) with ET_(30)_ for probes **(1)** and **(2)** in organic solvents.

The absorption data of probes in various solvents were analyzed with respect to different polarity scales. To do this, the absorption spectra were converted to the corresponding transition energy (ET = 28591.5/λ_max_ (nm)^35^. Figure 4 shows the relationship between the transition energy of the probes and ET_(30)_, which produced scattered plots. However, from the linearity of certain points in the scattered plots and the obtained slopes, the solvents can be classified into three distinct categories: aprotic, polar aprotic, and polar protic solvents.

For probe (1), the sensitivity trend of polarity towards the change in transition energy in each solvent category is as follows: polar aprotic (0.27) > polar protic (-0.072) > aprotic (-0.046). For probe (2), the trend is polar aprotic (0.895) > aprotic (0.167) > polar protic (0.052). The negligible contribution of protic solvents to the transition energy can be attributed to the strong hydrogen bonding between the hydrogen atoms of the protic solvents and the nitrogen and oxygen centers in the probes: five nitrogen atoms and one oxygen in probe (1), and five nitrogen atoms and three oxygen atoms in probe (2). As a result, the polar characteristics of the solvents are not effectively sensed by the dyes. Therefore, solute-solvent interaction is primarily driven by hydrogen bonding. In polar aprotic solvents, solute-solvent interaction is mainly influenced by the solvent’s polar characteristics, leading to the highest sensitivity in all probes.

A multi-parameter regression model has been employed for the quantitative assessment of solute-solvent interactions and absorption shifts, providing insights into the solvent effect on the investigated probes. Regression analysis was conducted on all solvent parameters to determine the contribution of each parameter to the ET values of the probe. According to the Kamlet-Abboud-Taft and Catalán models^36^, the relevant parameters include hydrogen bond donor ability (α), hydrogen bond acceptor ability (β), Taft’s polarity parameter (π*), solvent acidity (SA), solvent basicity (SB), and dipolarity/polarizability (SPP). Additionally, solvent parameters such as Reichardt’s single-parameter solvent polarity scale (E^N^_T_), refractive index (n), dielectric constant (D), solubility parameter (δ), and dipole moment (µ) considered to fully characterize solute-solvent interactions.

**Table:** 

Probe (1)	Probe (2)
ET_(Probe 1)_ = 76.52 + 3.42 α + 7.40 β + 8.50 π^∗^-3.25 SA-6.55 SB-29.75 SPP (*R* = 0.98)	ET_(Probe 2)_ = 70.01 + 0.04α + 4.13 β + 4.87 π^∗^-0.04 SA -8.22SB + 17.52SPP (*R* = 0.97)
ET_(Probe 1)_ = 70.57 + 3.94 E^N^_T_ − 0.135 µ -5.73 *n* − 0.043 D − 0.195 δ (*R* = 0.96)	ET_(Probe 2)_ = 61.80–4.53 E^N^_T_ + 0.16 µ + 7.24 n – 0.085 D + 0.502 δ (*R* = 0.97)

Multiple regression analysis of the ET values for probes (1) and (2) using these solvent parameters yielded the following models:

From the obtained models, it can be observed that the ET values of the probe are influenced by the cooperative effects of all modes of solvation. The large regression coefficients for Taft’s polarity parameter (π*), dipolarity/polarizability (SPP), Reichardt’s solvent polarity scale (E^N^_T_), and refractive index (n) confirm that solvent polarity, polarizability, and dispersion forces significantly affect the electronic absorption spectra of solutes. Furthermore, the high regression coefficient (R) indicates the robustness and predictive accuracy of the regression models. High π* and SPP coefficients indicate strong solute-solvent dipole interactions, stabilizing excited states and often causing bathochromic (red) shifts in π→π* and charge-transfer transitions^37^. A large E^N^_T_ coefficient further supports the influence of overall solvent polarity on spectral shifts, particularly in solvatochromic systems^38^. Meanwhile, a high regression coefficient for (n) suggests that electronic polarizability and dispersion forces modulate transition energies, affecting both peak positions and intensities^39^.

**Fig. 5:**
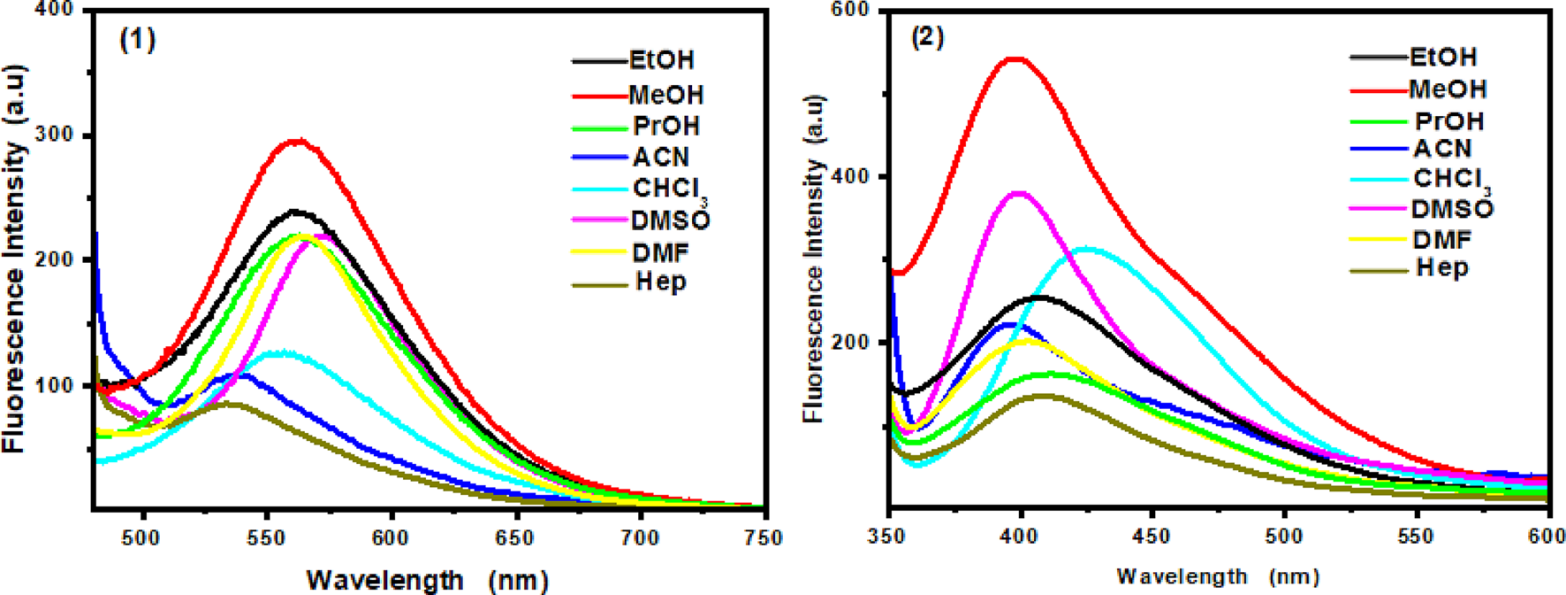
Fluorescence spectra of the investigated fluorescent probes **(1)** and **(2)** in the different solvents of various polarities.

Figure 5 shows the emission spectra of probes (1) and (2) in various solvents. The emission is more sensitive to solvent polarity than absorption, indicating that the excited singlet state is more polar than the ground state. Fluorescence intensity increases in polar solvents and is almost absent in the highly nonpolar Hep. For probe (1), the fluorescence intensity in MeOH was 3.5 times higher than in Hep. Increasing solvent polarity caused a notable red shift (27 nm) and band broadening, indicating stabilization of the highly dipolar excited state in polar solvents. The pronounced red shift, increased Stokes shifts, and broader emission bands with rising solvent polarity indicate a strong intramolecular charge transfer (ICT) character, with excited-state dipole moments higher than those of the ground state.

Analysis of the molecular structures of the probes reveals electron donor–acceptor pairs, which give rise to resonating structures through ICT as illustrated in Fig. 3. For probe (1), the resonance structure ‘A’ involves the delocalization of the lone pair of electrons on the oxygen atom of the hydroxyl group with the nitrogen atom of the azo group. In structure ‘B’, the electron density is donated from the azo group to the nitrogen of the triazole ring via tautomerism resulting in the transfer of the hydrogen atom. In probe (2), resonance structure ‘A’ is characterized by the lone pair of electrons on the nitrogen atom (NH) of the triazole moiety interacting with the nitrogen of the azo group. Structure ‘B’ shows the electron density donation from both the azo group and the hydroxyl group to the carboxylic group (due to the negative mesomeric effect (-M) of the carboxylic group and the positive mesomeric effect of the hydroxyl group (+ M)). The weak intensity in the fluorescence spectra of probe (2) can be attributed to the strong electron-withdrawing effect of the carboxylic group (-M). This weak fluorescence can be explained by efficient nonradiative decay processes, such as singlet-triplet intersystem crossing and internal conversion^40^.

**Fig. 6:**
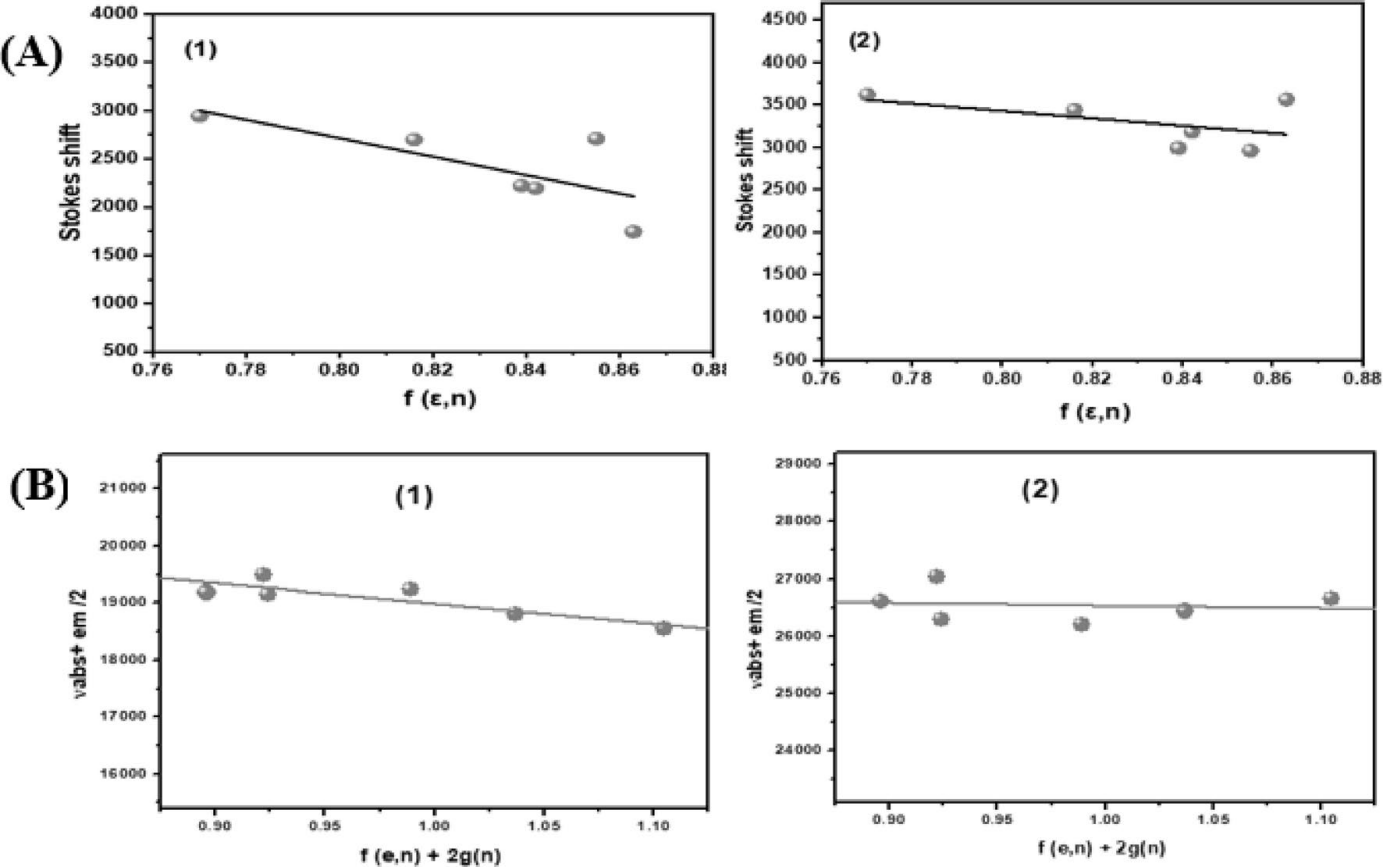
(**A**) Plots of Stokes shifts versus polarity *f* (*ε*,n), and (**B**) Plot (*v*_abs+em_) versus *f* (*ε*,n) + 2 g(n) for the probes (**1**) and (**2**) in different solvents.

The ground and excited-state dipole moments of probes (1) and (2) were determined using Bakhshiev’s and Kawski-Chamma-Viallet’s equations (excluding chloroform and heptane^37,38^. The cavity radius was taken as 40% of the distance between the two farthest atoms along the charge-separation axis^41^, determined after geometry optimization of probes (1) and (2) using ArgusLab 4.0 and HyperChem 8.03 (PM3 method), yielding 4.59 and 4.62 Å, respectively. The slopes m₁ and m₂ from the fitted Bakhshiev’s and Kawski-Chamma-Viallet equations (Fig. 6), along with the calculated dipole moments, are listed in Table 1. The calculated ground and excited dipole moments are 4.87 and 7.3 D for probe (1) and 10.7 and 9.42 D for probe (2), respectively, indicating higher polarization in the excited state. This reflects strong solute-solvent interactions and a relaxed excited state facilitated by ICT along the probe’s donor-acceptor pathways. This affirms too, the existence of a more relaxed excited state, due to ICT favored by the cooperative effects of the 8-hydroxy quinoline ring to the triazole moiety in s via the azo linkage.

**Table 1:** Maximum absorption and fluorescence wavelengths of the investigated fluorescent probes **(1)** and **(2)** in different solvents.

Fluorescent probe	(1)					(2)			
Solvent	λ^a^_max_ nm	λ^f^_max_ nm	ε Lmol^− 1^ cm^− 1^	E_T(30)_	Δ$\:\stackrel{-}{\boldsymbol{v}}$	λ^a^_max_ nm	λ^f^_max_ nm	ε Lmol^− 1^ cm^− 1^	Δ$\:\stackrel{-}{\boldsymbol{v}}$
MeOH	487	561	14,375	55.4	2708.56	356	398	43,333	2964.25
EtOH	488	562	12,250	51.9	2698.20	357	407	37,100	3441.18
PrOH	483	563	14,125	50.7	2941.94	357	410	46,666	3620.96
ACN	491	537	4000	45.6	1744.62	347	396	43,666	3565.91
DMSO	509	573	21,250	45.1	2194.35	354	399	44,466	3185.93
DMF	502	565	18,750	43.2	2221.20	358	401	42,000	2995.31
CHCl_3_	482	556	15,250	39.1	2761.27	355	425	46,000	4639.60
Hep	479	534	9875	31.1	2150.23	361	405	37,000	3009.47

### Metal ions sensing response

**Fig. 7:**
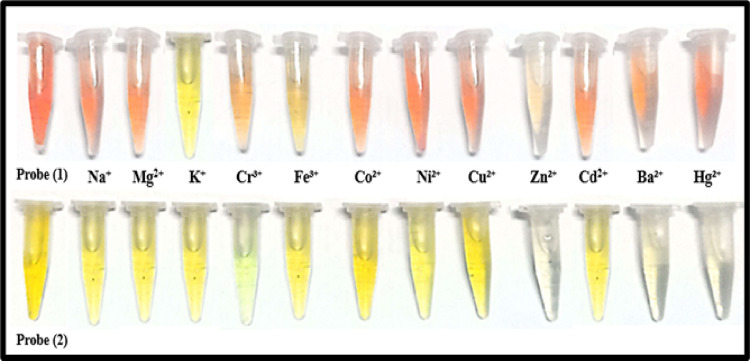
A selective visual color changes upon the addition of various metal ions to the investigated probes (**1**) and (**2**).

Probes (1) and (2) were evaluated as colorimetric sensors against various metal ions (Na⁺, Mg^2+^, K⁺, Cr^3+^, Fe^3+^, Co^2+^, Ni^2+^, Cu^2+^, Zn^2+^, Cd^2+^, Ba^2+^, and Hg^2+^) using preliminary chemosensory tests and spectrophotometric measurements. Probe (1) showed visible color changes from deep orange to yellow, brown, olive, pink, or pale yellow depending on the metal ion, while probe (2) changed from deep yellow to green, pale yellow, colorless, or yellow. No changes were observed with Na⁺, Mg^2+^, K^+^, and Cd^2+^ (Fig. 7), confirming that both probes can act as optical sensors through metal–ligand complex formation.

**Fig. 8:**
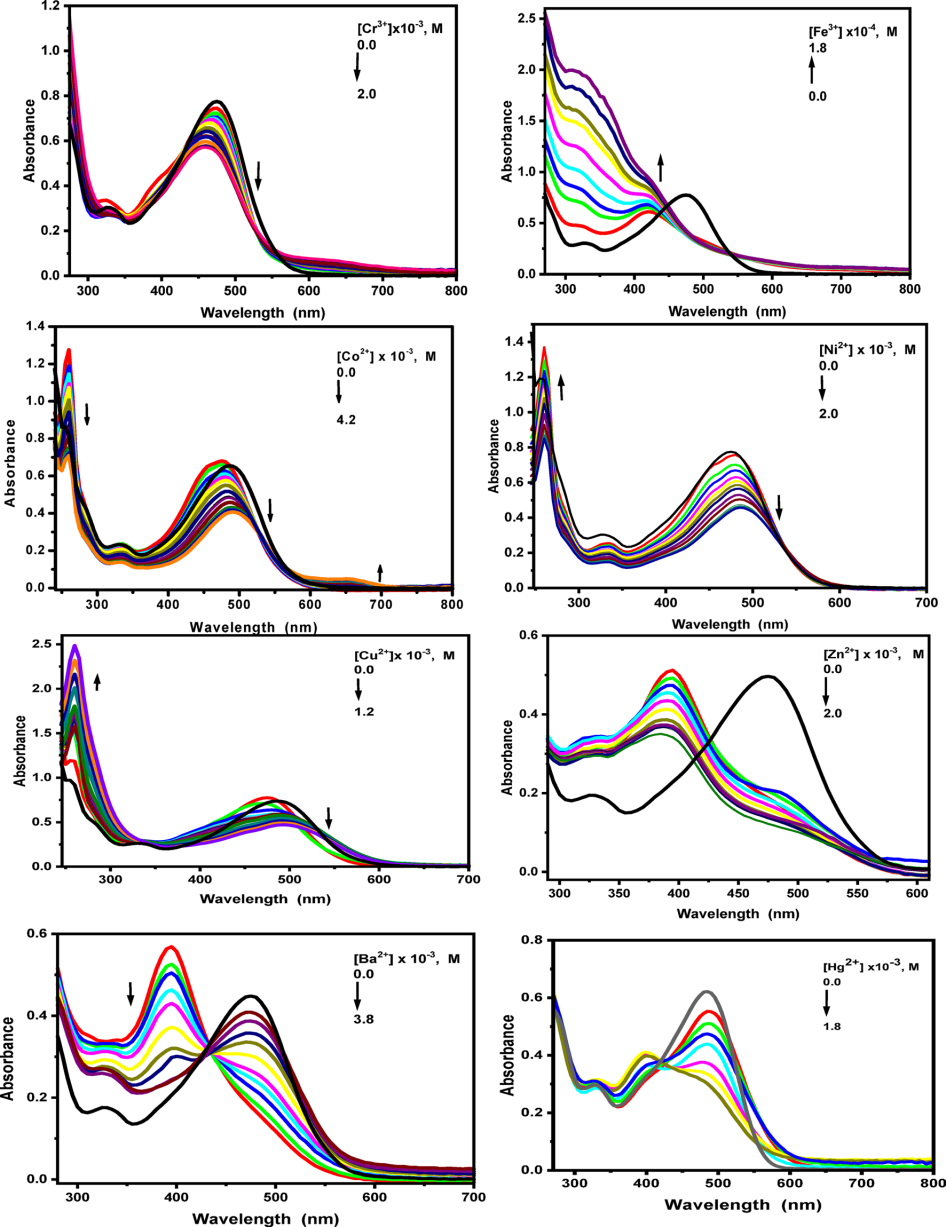
Absorption spectral changes of fluorescent probes **(1)** upon addition of different concentrations of the studied metal ions in ethanolic solution.

**Fig. 9:**
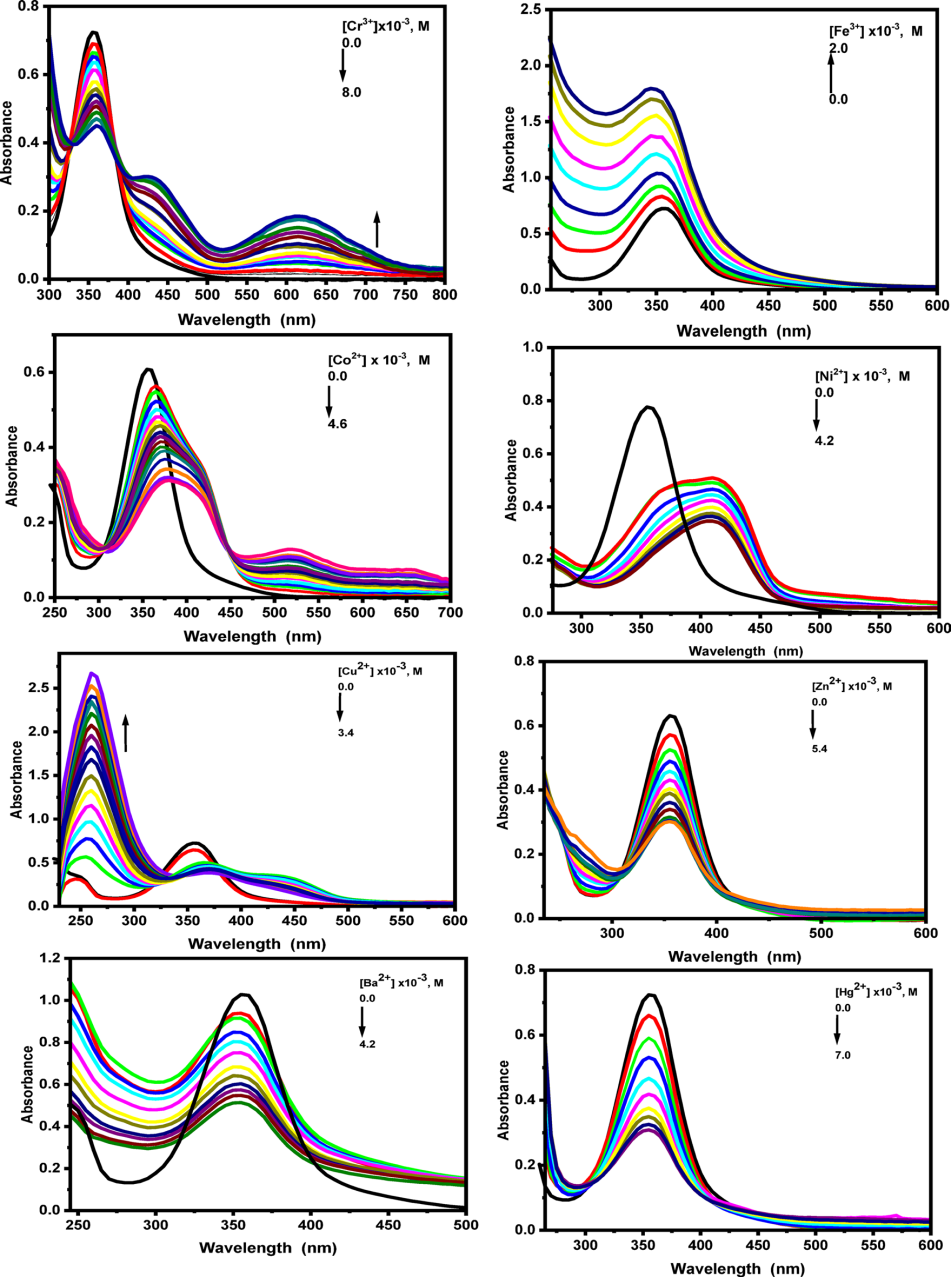
Absorption spectral changes of fluorescent probes **(2)** upon addition of different concentrations of the studied metal ions in ethanolic solution.

The sensing response of probes (1) and (2) towards the mentioned metal ions is quantitatively evaluated through absorption and emission spectral shifts. Figs. S6, S7 in the supplementary materials and Figs. 8 and 9 illustrate that both probes maintain high sensitivity toward the specified metal ions. In general, upon the addition of each metal ion to the probe solutions, the absorbance peaks of the free probes (1) and (2), located at 476 nm and 357 nm, respectively, decrease sharply. For probe (1), the absorption spectrum gradually decreases upon the addition of varying concentrations of Na^+^, Mg^2+^, and K^+^ ions. In the presence of Na^+^ ions (0–1.3 × 10⁻⁴ M), new well-defined bands appear at 399 and 488 nm, accompanied by the formation of an isosbestic point at 546 nm. For Mg^2+^ ions (0-2.4 × 10^− 4^ M), a hypsochromic shift is observed at 470 nm. In contrast, upon addition of K^+^ ions, a new absorption band emerges at 397 nm, along with two isosbestic points at 430 and 565 nm.

Upon the addition of varying concentrations of Cr³⁺ ions (0–2 × 10⁻³ M), the absorption spectrum of probe (**1**) gradually decreases, accompanied by a hypsochromic shift of 17 nm. Additionally, shoulder bands appear at 628 nm, and isosbestic point form at 530 nm. In the presence of higher concentrations of Ni^2+^ ions, a bathochromic shift of 10 nm in the absorption maximum is observed, with an isosbestic band at 539 nm. In contrast, the absorption spectra of probe (1) increase gradually with the addition of varying concentrations of Fe^3+^ ions (0 to 1.8 × 10^− 4^ M), resulting in new bands at 418 nm and 318 nm.

At lower concentrations of Co^2+^ and Cu^2+^ ions, blue-shifted bands appear at 474 nm and 473 nm, respectively. At higher concentrations, red-shifted bands are formed at 490 nm and 498 nm, with the appearance of a new visible band at 652 nm for Co^2+^ and isosbestic points at 538 nm and 543 nm for Cu^2+^ ions.

Upon interaction of probe (1) with Zn^2+^ ions, the absorption intensity at the band maxima decreases, accompanied by the appearance of new structured bands at 393 and 478 nm. In the case of Cd^2+^ ions, no significant change is observed in the absorption maximum, although an isosbestic point is formed at 568 nm. Upon addition of Hg^2+^ ions, the absorption spectrum decreases and exhibits a bathochromic shift of approximately 5 nm. At higher Hg^2+^ concentrations, a structured band emerges at 400 nm, along with the formation of an isosbestic point at 548 nm. Furthermore, when increasing concentrations of Ba^2+^ ions are introduced, the absorption spectra of probe (1) gradually decrease, with a new band appearing at 394 nm and an isosbestic point forming at 422 nm. For probe (2), upon the addition of various concentrations of Na^+^, Mg^2+^, and K^+^ ions, the absorption spectra gradually decrease. In the presence of Na^+^ ions (0-1.3 × 10^− 4^ M), two isosbestic points are observed at 326 and 398 nm, whereas with K⁺ ions, a single isosbestic point appears at 322 nm. For Mg^2+^ and Cd^2+^ ions, the absorption spectra decrease without any noticeable shift in the absorption maximum. In the case of Cr^3+^ ions (0–8 × 10^− 3^ M), the absorption spectra also decrease progressively, accompanied by the emergence of new bands at 429 and 615 nm, along with two isosbestic points at 325 and 388 nm. The UV–Vis spectra of probe (2) in the presence of Fe^3+^ ions exhibit a hyperchromic effect at 345 nm. Moreover, upon addition of increasing concentrations of Co^2+^ ions (0-4.6 × 10^− 3^ M), the absorption spectra decrease, with a redshifted band appearing at 379 nm, two additional bands emerging at 520 and 655 nm, and two isosbestic points forming at 306 and 446 nm. When different concentrations of Ni^2+^ ions are added to the probe (2) solution, the absorption bands gradually decrease, with the formation of new bands at 411 nm and 462 nm. Furthermore, for the interaction of probe (2) with Cu^2+^ ions, the absorption at the band maximum (357 nm) increases, with the appearance of two bands at 364 nm and 438 nm, and the formation of an isosbestic point at 334 nm.

Upon adding varying concentrations of Zn^2+^ and Hg^2+^ ions, the absorption spectrum of probe (2) decreases, with the formation of two isosbestic points at 308 nm and 410 nm, and at 304 nm and 452 nm, respectively. Finally, gradual addition of Ba^2+^ ions (0-4.2 × 10^− 3^ M) results in a smaller response in the absorption band, shifting from 357 nm to 354 nm, with a decrease in absorption intensity.

The notable, fast color, and spectral changes upon addition of the utilized metal ions indicate that the investigated probes might be potentially as selective sensors in environmental monitoring, biomedical applications, and industrial analysis.

**Fig. 10:**
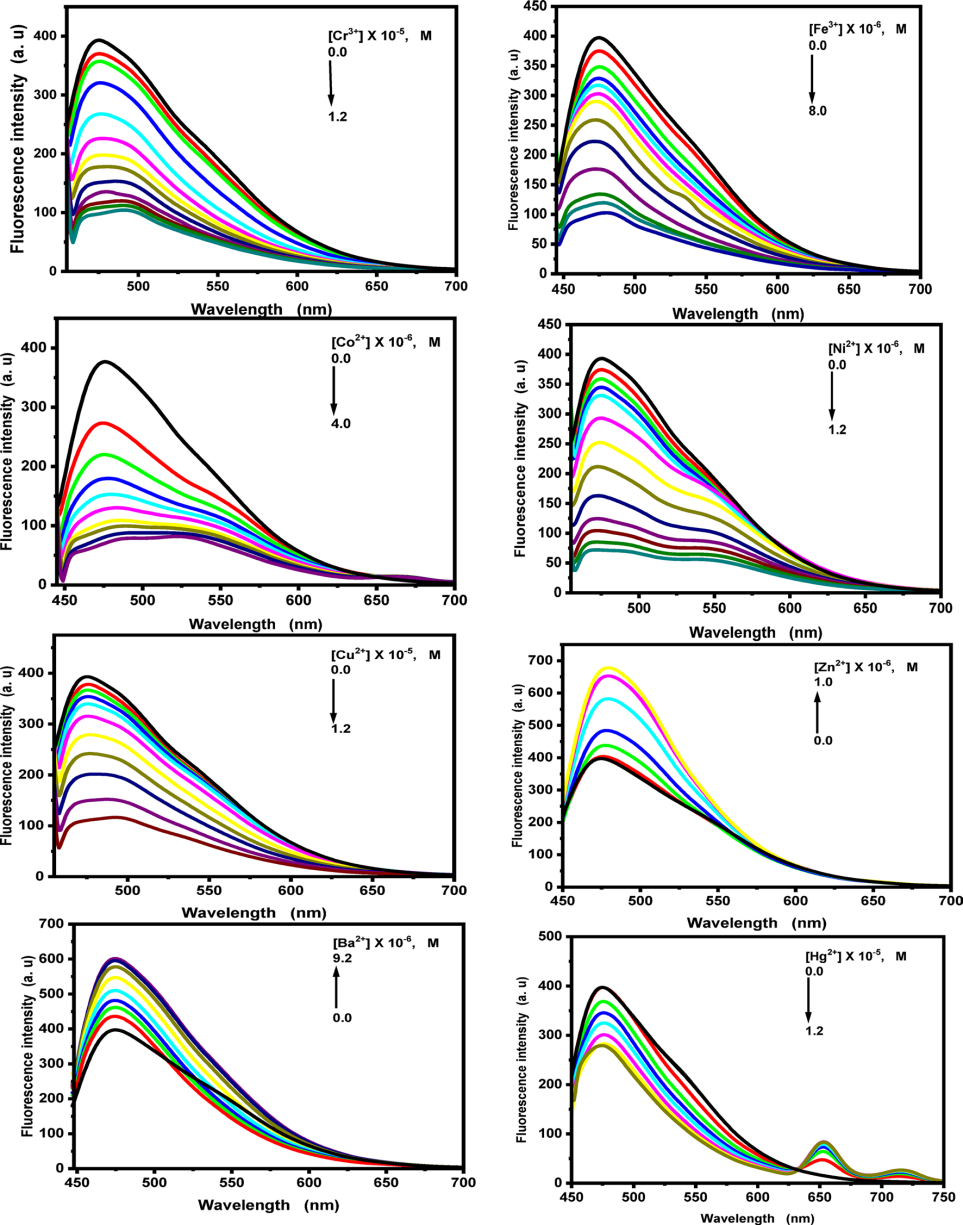
Fluorescence spectra of fluorescent probe **(1)** upon addition of different concentrations of the studied metal ions in ethanolic solution.

**Fig. 11:**
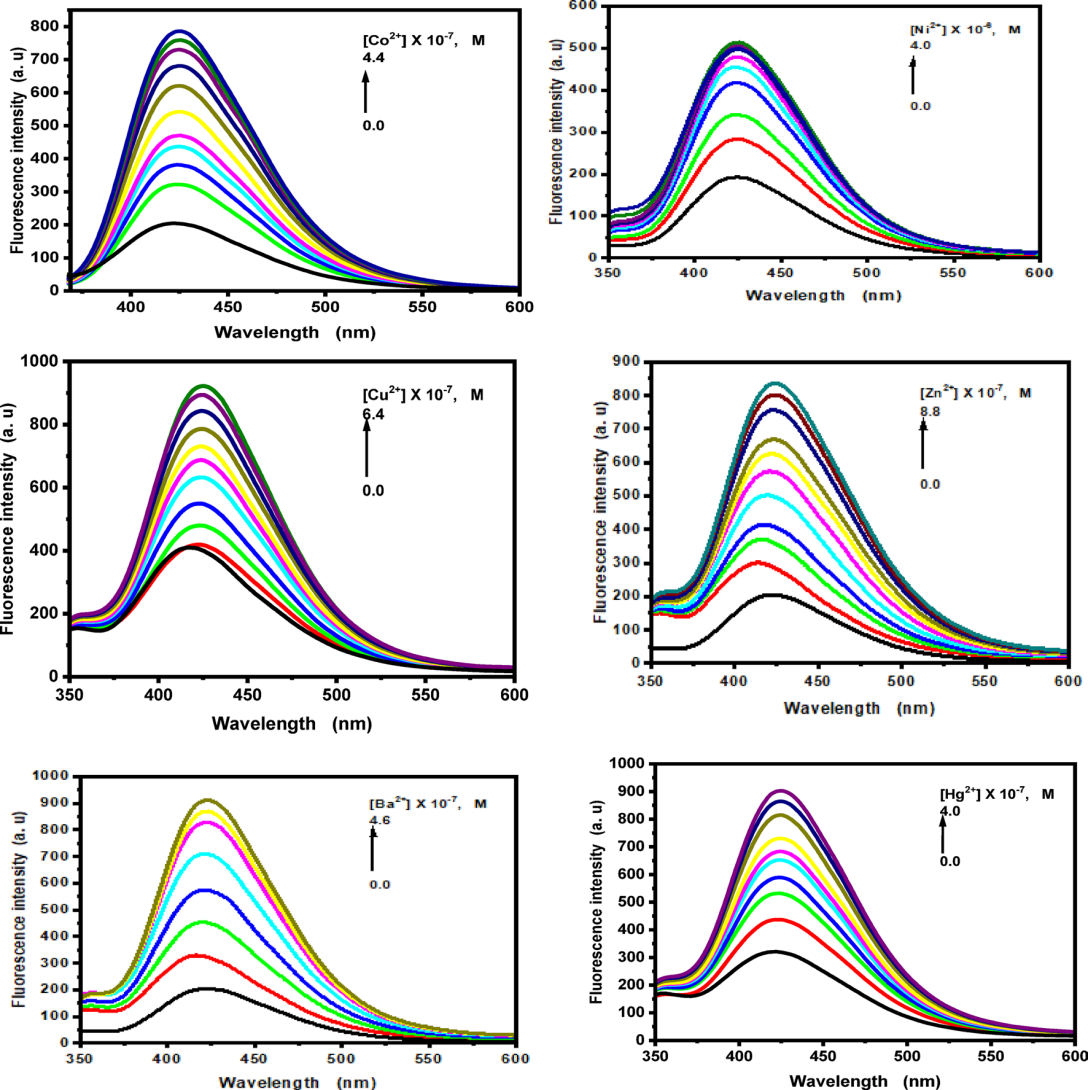
Fluorescence spectra of fluorescent probe **(2**) upon addition of different concentrations of the studied metal ions in ethanolic solution.

The probes’s sensitivity to the metal ions Na^+^, Mg^2+^, K^+^, Cr^3+^, Fe^3+^, Co^2+^, Ni^2+^, Cu^2+^, Zn^2+^, Cd^2+^, Ba^2+^, and Hg^2+^ was also assessed by looking at the fluorescence activity of the probes (1) and (2) in the presence of the metal ions (Figs. 10 and 11, and Figs. S8, S9 in the supplementary materials). In general, the fluorescence response of probe (1) markedly decreased upon the addition of various concentrations of Cr³⁺, Fe³⁺, Co^2+^, Ni^2+^, Cu^2+^, and Hg^2+^ ions, whereas the fluorescence intensity increased progressively with the addition of Na^+^, Mg^2+^, K⁺, Zn^2+^, Cd^2+^, and Ba^2+^ ions. For Na^+^, Mg^2+^, K^+^, and Cd^2+^ ions, the fluorescence spectra exhibited hypsochromic shifts at 473, 454, 456, and 473 nm, respectively. In contrast, for Cr^3+^ and Cu^2+^ ions, notable bathochromic shifts of 14 and 16 nm were observed. However, upon addition of Fe^3+^, Zn^2+^, and Ba^2+^ ions, no shift in the fluorescence maximum was detected. In the presence of Co^2+^ ions, a broad emission band appeared at 524 nm, whereas the addition of Ni²⁺ ions resulted in the formation of structured bands at 471 and 549 nm. Furthermore, when increasing concentrations of Hg^2+^ ions (0-1.2 × 10^− 5^ M) were added, new emission bands emerged at 653 and 716 nm.

For probe (2), the fluorescence band, centered at 421 nm in ethanolic solution, increased progressively upon the addition of various concentrations of Na^+^, Mg^2+^, K^+^, Cr^3+^, Fe^3+^, Co^2+^, Ni^2+^, Cu^2+^, Zn^2+^, Cd^2+^, Ba^2+^, and Hg^2+^ ions. A bathochromic shift in the emission maximum was observed for Cr^3+^, Fe^3+^, Co^2+^, Ni^2+^, Cu^2+^, Zn^2+^, Ba^2+^, and Hg^2+^ ions, while a hypsochromic shift to 417 nm occurred in the presence of Na^+^ ions. In contrast, no significant shift in the emission maximum was observed for Mg^2+^, Cd^2+^, and K^+^ ions. These spectral variations confirm the complexation of probe (2) with the aforementioned metal ions.

**Fig. 12:**
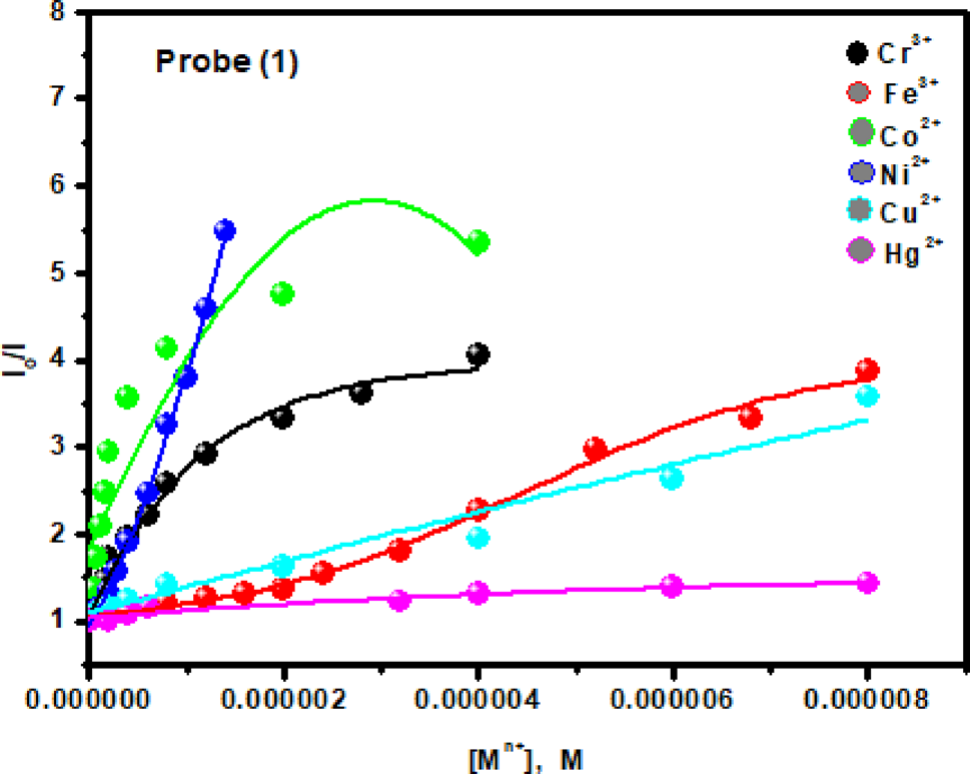
The Stern-Volmer plots for the quenching of the probe (**1**) by the mentioned metal ions.

The fluorescence quenching mechanism for the quenching of the probe (1) by Fe^3+^, Co^2+^, Ni^2+^, Cu^2+^ and Hg^2+^ ions can be analyzed with the help of the Stern-Volmer equation^42^, where; *I*_*o*_ */I* = 1 + *K*_*SV*_[*M*^*n*+^], where I_o_ and I are the fluorescence emission intensities of the investigated probe in the absence and presence of different concentrations of metal ions. K_SV_ and [M^n+^] are the Stern-Volmer quenching constant and the metal ions concentration. The Stern–Volmer plots (Fig. 12) shows upward/downward curvatures at higher metal ion concentrations, confirming static quenching due to the formation of a ground-state non-fluorescent probe–metal complex.

The modified Stern–Volmer Eq. (1)^43^ was used to calculate K_sv_ as follows;

1 $$\:\frac{{\mathrm{F}}_{\mathrm{o}}}{{\mathrm{F}}_{\mathrm{o}}-\mathrm{F}}=\frac{1}{{{f}_{a}\:\mathrm{K}}_{\mathrm{S}\mathrm{V}}{[\mathrm{M}}^{n+}]}+\frac{1}{{f}_{a}}\:\:\:\:$$

**Fig. 13:**
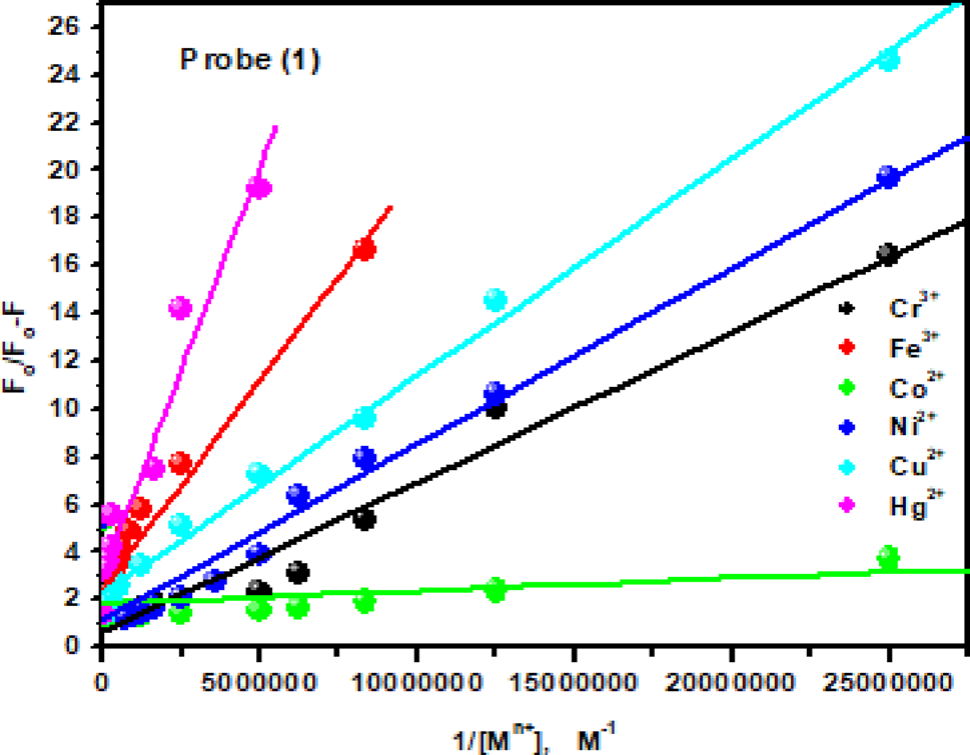
The modified Stern-Volmer plots for fluorescence quenching of the probe (**1**) by the mentioned metal ions.

F_o_ and F are the relative fluorescence intensities of the probes in the absence and presence of the metal ions, respectively; *f*_a_ is the fraction of fluorophore accessible to the quencher; [M^n+^] is the concentration of the metal ions, Fig. 13. The calculated K_sv_ values are summarized in Table 2, showing that Co^2+^ exhibits the strongest quenching effect among the tested metal ions, confirming that probe (1) has high specificity and sensitivity toward Co^2+^.

A Job plot analysis of the interaction between probes 1 and 2 with Co²⁺ ions revealed a 1:1 binding stoichiometry (Fig. S10). Accordingly, the Job plot results support the formation of a 1:1 probe-Co²⁺ complex and a corresponding coordination structure is therefore proposed.

The binding constant (K_b_) of the complexation between the investigated probes and the studied metal ions (1:1 probe-Co^2+^ complex) can be calculated from the slope of Benesi–Hildebrand plot using the following Eq. (2)^44^

2 $$1/\mathrm{F} -\mathrm{F}_{\text o} = 1/\text {F}_{\infty}-\mathrm{F}_{\text o}+(1/\mathrm{Kb}[\mathrm{M}^{n+}])(1/\text {F}_{\infty}-\mathrm{F}_{\text o})$$

**Fig. 14:**
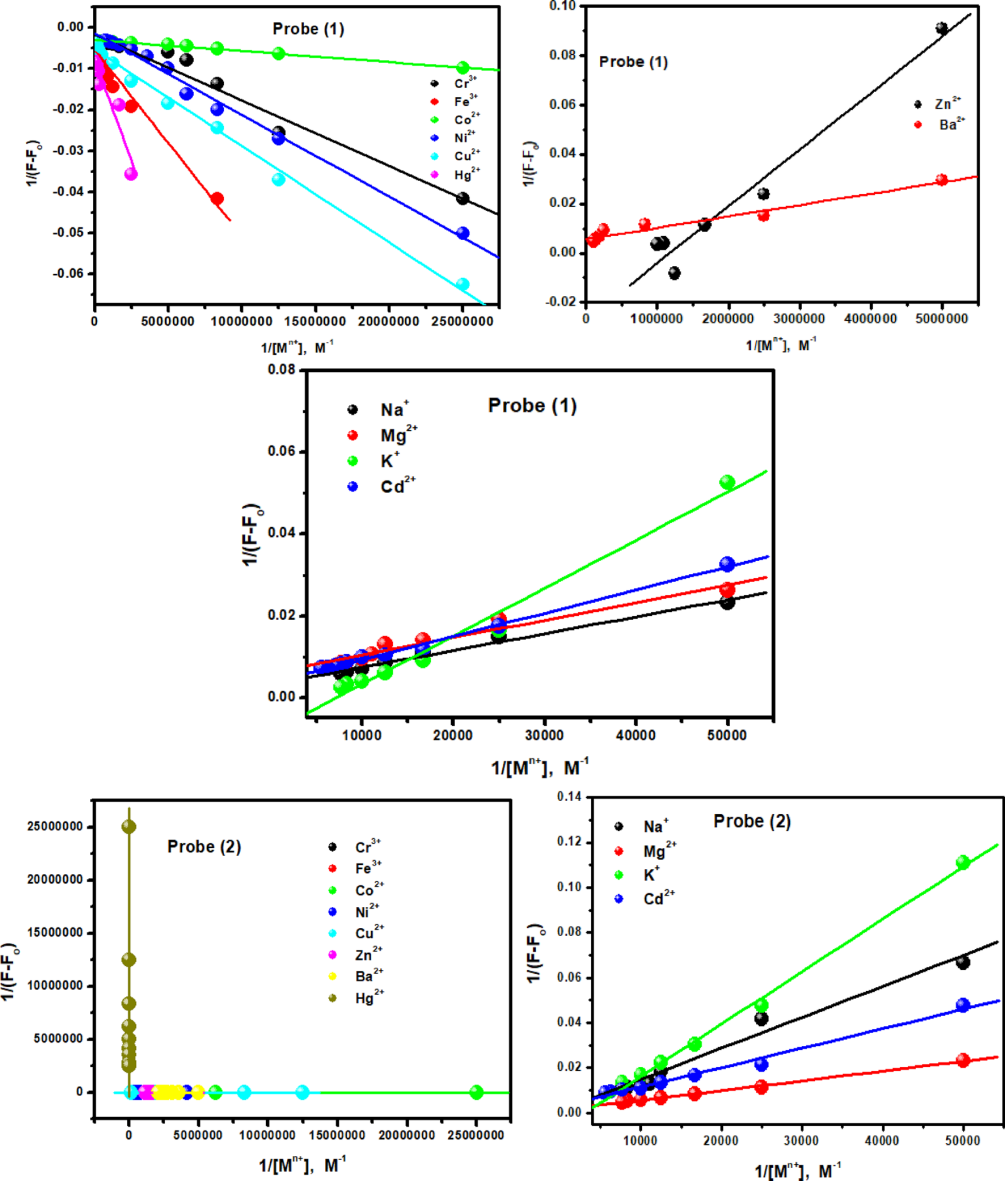
Benesi–Hildebrand plots from fluorescence titration data of the investigated probes (**1**) and (**2**) with the studied metal ions.

Here F is the observed fluorescence at each concentration tested, F_o_ is the fluorescence intensity of analyte in the absence of metal ion, and [M^n+^] is the concentration of metal ion. From the plot of 1/F-F_o_ against 1/[M^n+^], the value of K_b_ has been determined from the slope (Fig. 14) and summarized in Table 2. A cursory glance at the data indicates that probe (1) exhibits stronger chelation with most metal ions than probe (2) as reflected by higher K_b_ values, except for Ni^2+^. Co^2+^ shows the highest binding constant, attributed to its strong chelation with both probes (1) and (2).

Additionally, the limit of detection (LOD) andlimit of quantitation (LOQ) of the probes (1), (2) were estimated according to the following Eq. (3)^45^

3 $$\mathrm{LOD}=\mathrm{3}\, \mathrm{S} / \mathrm{m}, \mathrm{LOQ}=10 \,\mathrm{S} / \mathrm{m}$$

where S and m are the standard deviation and the slope of the fluorescence titration data based on a reported and widely used method^45^. Linear regression curves were fitted, the LOD and LOQ were calculated, as summarized in Table 2. The detection limits obtained for the studied metal ions using the investigated probes exceeded WHO drinking water guidelines^46^ but were lower than those reported for many other organic probes^47–55^. Table 3 compares several key metrics of recently reported materials and current research. Notably, the investigated probes can detect a wide range of metal ions (Na^+^, Mg^2+^, K^+^, Cr^3+^, Fe^3+^, Co^2+^, Ni^2+^, Cu^2+^, Zn^2+^, Cd^2+^, Ba^2+^, and Hg^2+^) through significant changes in absorption and emission spectra (quantitative analysis) and observable color changes (qualitative colorimetric response).

**Fig. 15:**
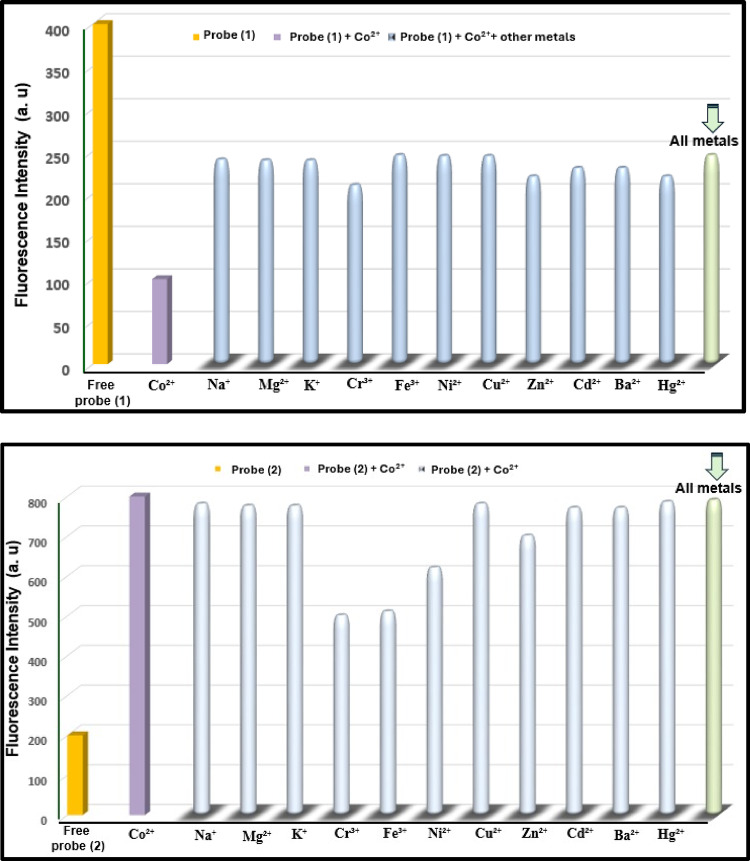
Selectivity of the investigated probes (**1**) and (**2**) to Co^2+^ ions over the different metal ions in ethanolic solution at 4 × 10^− 7^ M.

The selectivity of the investigated probes in EtOH towards various metal ions (Na^+^, Mg^2+^, K^+^, Cr^3+^, Fe^3+^, Co^2+^, Ni^2+^, Cu^2+^, Zn^2+^, Cd^2+^, Ba^2+^, and Hg^2+^) was evaluated. To confirm the high selectivity for Co^2+^, competitive experiments were performed in the presence of the other metal ions. In a ternary mixture containing probes (1) and (2) (8 × 10^− 5^ and 3 × 10^− 5^ M, respectively), Co^2+^ (4 × 10^− 7^ M), and interfering ions (4 × 10^− 7^ M), the fluorescence intensity of the probe-Co^2+^ complex showed negligible changes. This indicates that the presence of other metal ions had no significant effect on the fluorescence response, confirming the probes’ strong selectivity for Co^2+^ (Fig. 15).

While the synthesized probes demonstrated remarkable solvatochromic behavior, strong Co^2+^ selectivity, and promising antibacterial activity, certain limitations should be acknowledged. Although binding stoichiometry and interaction mechanisms were supported by spectroscopic analyses (Job plot, Stern-Volmer, and Benesi-Hildebrand models), definitive structural elucidation of the probe-metal complexes through single crystal X-ray diffraction or advanced density functional theory calculations were not performed. In addition, key parameters relevant to practical sensor deployment such as pH tolerance, reversibility, recyclability, long-term stability, and photostability were not systematically investigated. The antibacterial evaluation was limited to agar diffusion assays, and further biological assessment including minimum inhibitory concentration, cytotoxicity profiling, and mechanistic studies are necessary.

Furthermore, although the triazole azo framework is known to exhibit diverse pharmacological activities, including potential anticancer properties, the antitumor activity of the synthesized probes and their metal complexes have not yet been explored. Given the observed metal-binding capability and structural features that may facilitate biomolecular interactions, future investigations will extend toward in vitro cytotoxicity screening against cancer cell lines and evaluation of the probe-metal complexes as potential antitumor agents. Accordingly, future work will focus on structural confirmation, computational modeling, expanded biological evaluation, and exploration of therapeutic potential to fully establish the environmental and biomedical applicability of these triazole based fluorescent systems.

**Table 2:** Spectral data, ionic radius, maximum absorption and fluorescence wavelengths, binding constants in both ground and excited states and stern-Volmer constants by the complexation of the investigated fluorescent probes **(1)** and **(2)** with the metal ions in EtOH.

Probe	(1)						(2)					
	λ^a^_max_ nm	λ^f^_max_ nm	K_sv_ × 10^6^ (M^− 1^)	K_b_ × 10^5^ (M^− 1^)	LOD × 10^− 6^ (M^− 1^)	LOQ × 10^− 6^ (M^− 1^)	λ^a^_max_ nm	λ^f^_max_ nm	K_sv_ (M^− 1^)	K_b_ × 10^5^ (M^− 1^)	LOD × 10^− 6^ (M^− 1^)	LOQ × 10^− 6^ (M^− 1^)
Na^+^	399, 488(476)*	473 (479)*	–	0.081	16.3	54.0	357 (357)*	417 (421 )*	–	0.009	31.7	105.9
Mg^2+^	470	454	–	0.15	27.5	91.8	357	421	–	0.03	22.6	76.0
K^+^	397	456	–	0.072	34.0	113.9	357	421	–	0.03	6.91	23.0
Cr^3+^	459	493	–	10.69	3.27	10.9	429,615	425	–	1.47	1.18	3.94
Fe^3+^	318,418	481	1.43	11.92	1.94	6.48	345	423	–	0.075	3.97	13.21
Co^2+^	652	524	35.2	113.48	0.58	19.29	379,520,655	427	–	46.0	0.06	0.20
Ni^2+^	486	471,549	1.57	7.10	0.47	1.56	411,462	426	–	17.0	4.48	14.90
Cu^2+^	473,498	495	2.50	22.57	2.75	9.17	364,438	426	–	12.0	8.31	27.70
Zn^2+^	393,478	481	–	8.69	1.87	6.23	457	425	–	7.3	0.10	0.34
Cd^2+^	476	473	–	0.065	58.6	195.3	357	421		0.034	55.9	186.0
Ba^2+^	394	475	–	17.35	3.17	10.56	354	425	–	16.4	0.16	0.54
Hg^2+^	400, 481	653,716	0.91	7.93	3.88	12.95	457	423	–	0.001	0.085	0.28

**Table 3:** A few aspects of some recently published related probes.

Entry	Probe	Metal ions	LOD	Binding constants (M^− 1^)	Applications	Ref
1	Fluorescent azo benzene-based probe (4′-hydroxyl-2,4-diaminoazobenzene, MP)	Al^3+^ and Fe^3+^	20µM–2.0 mM	6.80 × 10^4^ and 7.46 × 10^4^	Sensing and selectivity	^45^
2	Phenanthro[9,10-*d*]imidazole-coumarin-based Schiff bases	Fe^3+^	4.28 µM	1.52 × 10^5^	Sensing and selectivity	^46^
3	Salicylal derived Schiff base sensor	Co^2+^	0.782 µM	–	Sensing and selectivity	^47^
4	3-(-(2-hydroxyphenylimino) methyl)-4 H-chromen-4-one	Cu^2+^, Fe^3+^, and V^5+^	7.03, 5.16, 5.94 *µ*M	1.37 × 10^4^, 2.01 × 10^4^, 1.82 × 10^4^	Sensing	^48^
5	Tris(2-pyridinecarboxaldehyde)triaminoguanidinium chloride	Zn^2+^	2.5 × 10 − 6 M	1.9 × 10^5^	Sensing and selectivity	^49^
6	(E)-4,4’,4’’-(20-(4-(2-(2-hydroxybenzylidene)hydrazine-1-carbonyl)phenyl)porphyrin-5,10,15-triyl)tris(1-methylpyridin-1-ium)	Hg^2+^	17.3 µM	–	sensing	^50^
7	Rhodanine-Based Azo Dyes	Fe^3+^	5.14 µM	4.63 × 10^8^ M^− 1^	Sensing and selectivity	^51^
8	Pyrene-based chemosensor	Fe^3+^	–	1.27 × 10^4^	Biological study	^52^
9	A salicylaldehyde derived Schiff base *N*,* N*′- bis (p-chloro salicylidene)-1, 2- ethylenediamine	Zn^2+^	3.21 µM	6.17 × 10^9^	Biological study	^53^

### Antibacterial activity of the investigated probes (1) and (2)

**Fig. 16:**
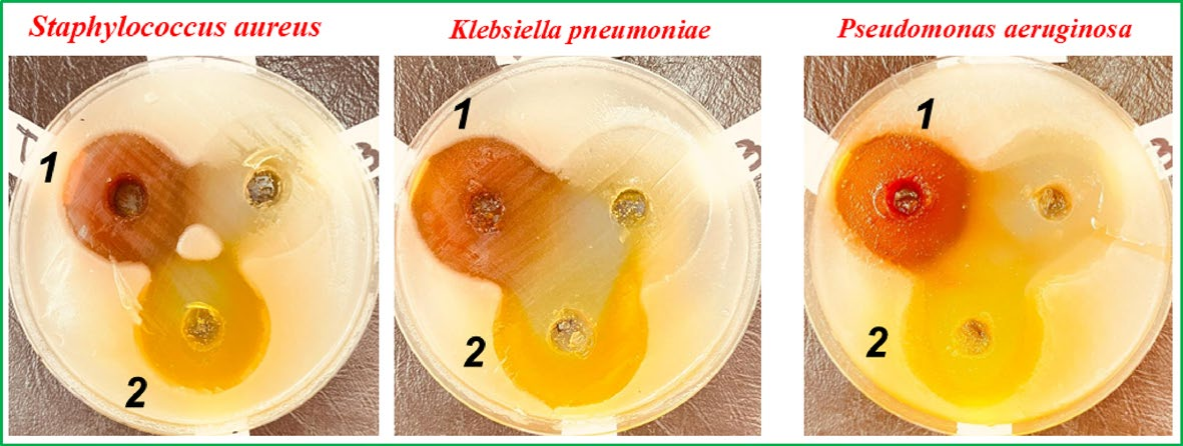
Antibacterial images of the investigated probes (**1)** and (**2)**.

Table 4; Fig. 16 present the antibacterial activity of the investigated probes (1) and (2), in comparison to ciprofloxacin against three bacterial strains: *Staphylococcus aureus*, *Pseudomonas aeruginosa*, and *Klebsiella pneumoniae*. The inhibition zone diameters (in mm) indicate the effectiveness of the compounds in inhibiting bacterial growth. Both probes(1) and (2) show a strong antibacterial effect, with inhibition zones larger than those of ciprofloxacin in all three bacterial strains tested. Probe (2) demonstrates slightly superior antibacterial activity, with inhibition zones consistently larger than probe (1) by up to 2.67 mm. For instance, against *Staphylococcus aureus*, probe (2) shows an inhibition zone of 34.67 mm, compared to probe (1) (34.00 mm). All three bacterial strains exhibit significant susceptibility to both probes, with *Klebsiella pneumoniae* showing the largest inhibition zones (39.67 mm for probe (2)), suggesting that this strain is particularly sensitive to these compounds. The inhibition zones for *Pseudomonas aeruginosa* are relatively similar for both compounds, ranging from 33.00 mm to 34.67 mm. The results suggest that probes (1) and (2) may be effective candidates for further investigation in antibacterial therapy, particularly against multidrug-resistant bacteria like *Staphylococcus aureus* and *Pseudomonas aeruginosa*.

**Table 4:** Antibacterial inhibition zone diameters (mm) of the probes (**1**) and (**2**) aginst some bacterial starins.

Cpd.	Staphylococcus aureus	Pseudomonas aeruginosa	Klebsiella pneumoniae
	Inhibition zone (mm)		
Ciprofloxacin	19 ± 0.35	24 ± 0.40	21 ± 0.25
Probe (1)	34.00 ± 1.0	33.00 ± 1.0	37.00 ± 2.65
Probe (2)	34.67 ± 0.58	34.67 ± 0.58	39.67 ± 0.58

## Conclusion

In summary, two newly synthesized triazole-based fluorescent probes demonstrated favorable photophysical properties, including strong solvent polarity dependence and enhanced dipole moments in the excited state. Both probes exhibited high selectivity and sensitivity for Co²⁺ ions, with detection limits well below the safety thresholds set by the EPA and WHO, highlighting their potential for environmental monitoring applications. Additionally, the strong binding affinity toward Co^2+^ reinforces their utility as selective metal ion sensors. Beyond sensing capabilities, the probes also showed notable antibacterial activity, particularly against multidrug-resistant *Staphylococcus aureus*, *Pseudomonas aeruginosa*, and *Klebsiella pneumoniae* suggesting their promise as dual-function materials for both chemical sensing and antimicrobial applications. 

## Supplementary Information

Below is the link to the electronic supplementary material.

Supplementary Material 1

## Data Availability

All data generated or analyzed during this study are included within the manuscript.
