# Supplementary material for: Synthesis and optical behaviors of novel triazole fluorescent probes involving solvatochromic behavior, metal ions detection and their antibacterial activity
Source: Sci Rep. 2026 Apr 7;16:11663. doi: 10.1038/s41598-026-41364-y (PMC13061940; doi:10.1038/s41598-026-41364-y)
Supplement: Supplementary file 1 — Supplementary Material 1 [file 41598_2026_41364_MOESM1_ESM.docx]

**Synthesis and Optical Behaviors of Novel Triazole Fluorescent Probes Involving Solvatochromic Behavior, Metal Ions Detection and their Biological Activity**

Hazem M. Elkholy*, Wafaa M. Hamada, Marwa N. El-Nahass

Chemistry Department, Faculty of Science, Tanta University, Tanta 31527, Egypt.

^*^Corresponding author: hazem_elkholy@science.tanta.edu.eg

**Fig. S1**: FT-IR spectra of the probes **(1)** and **(2)**.


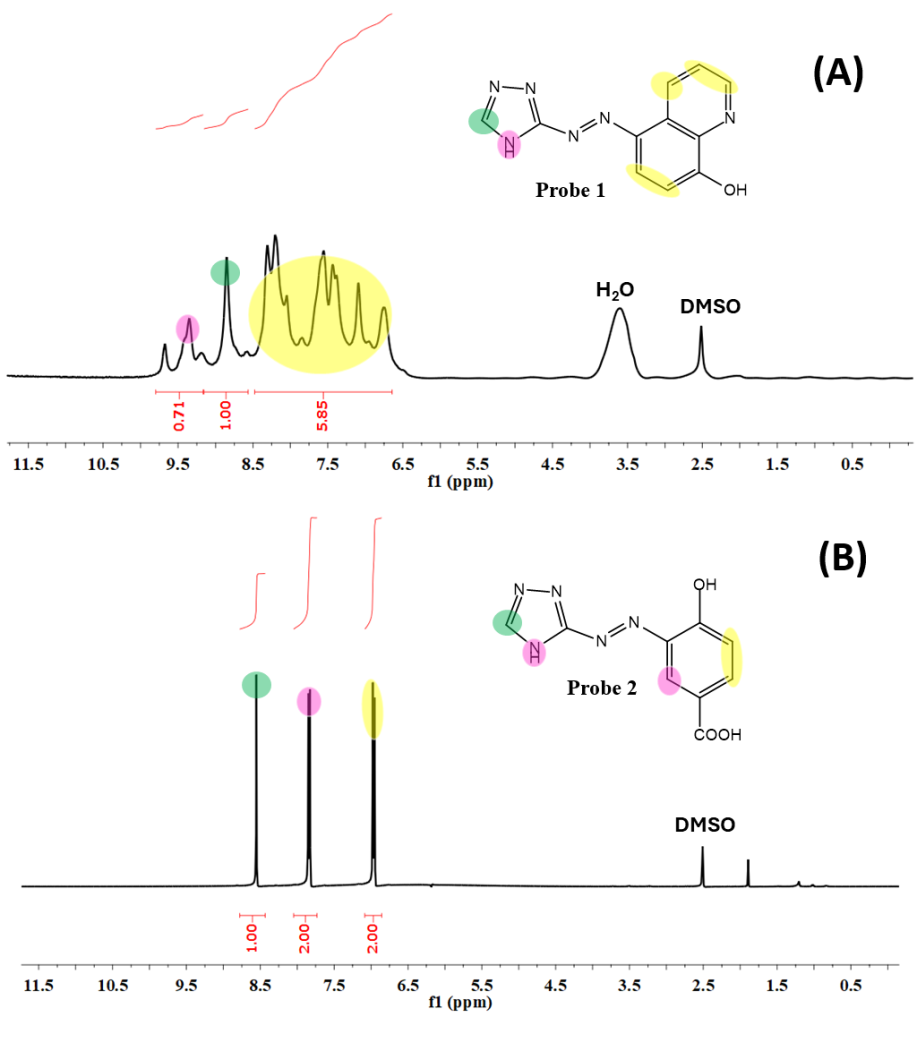


**Fig. S2**: ^1^H-NMR spectra of probes **(1)** and **(2)**.


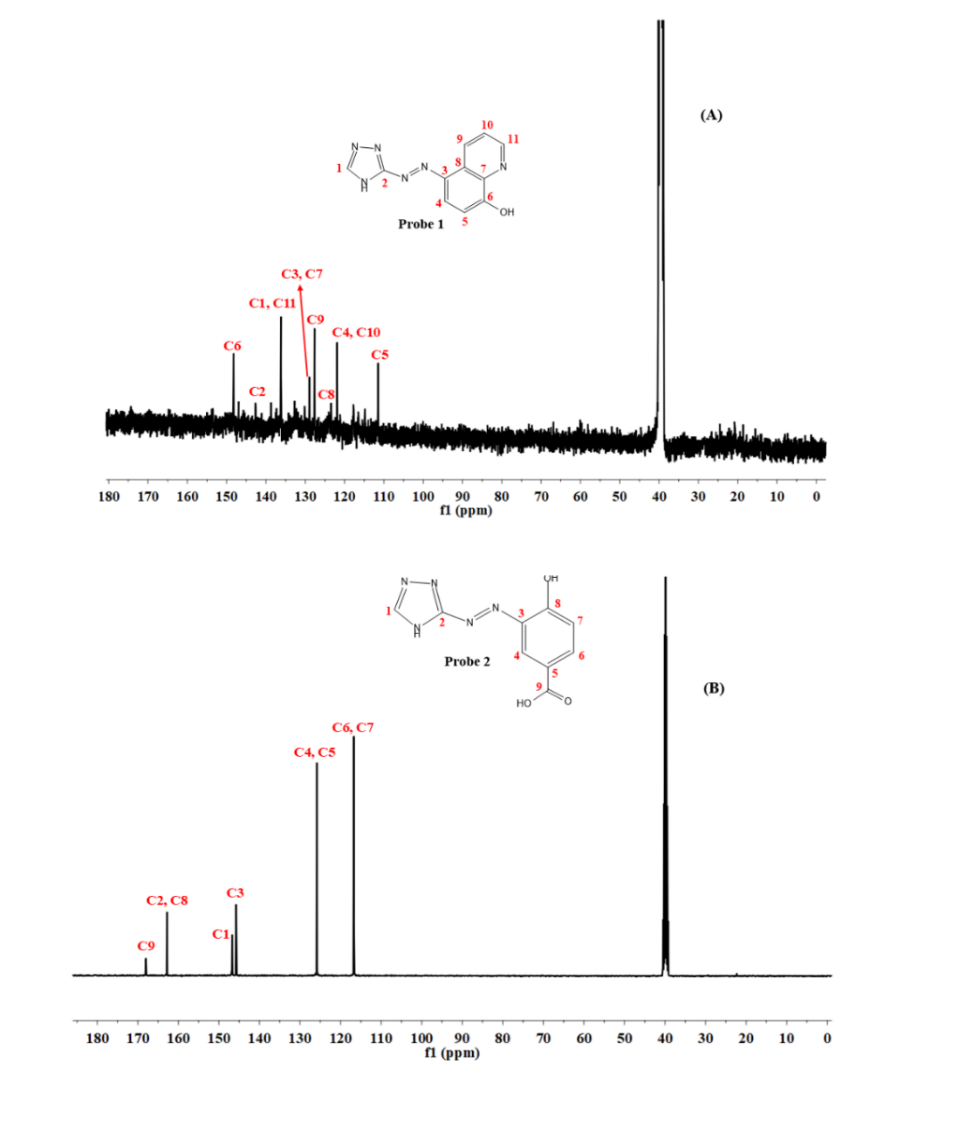


**Fig. S3**: ^13^C-NMR spectra of probes **(1)** and **(2)**.


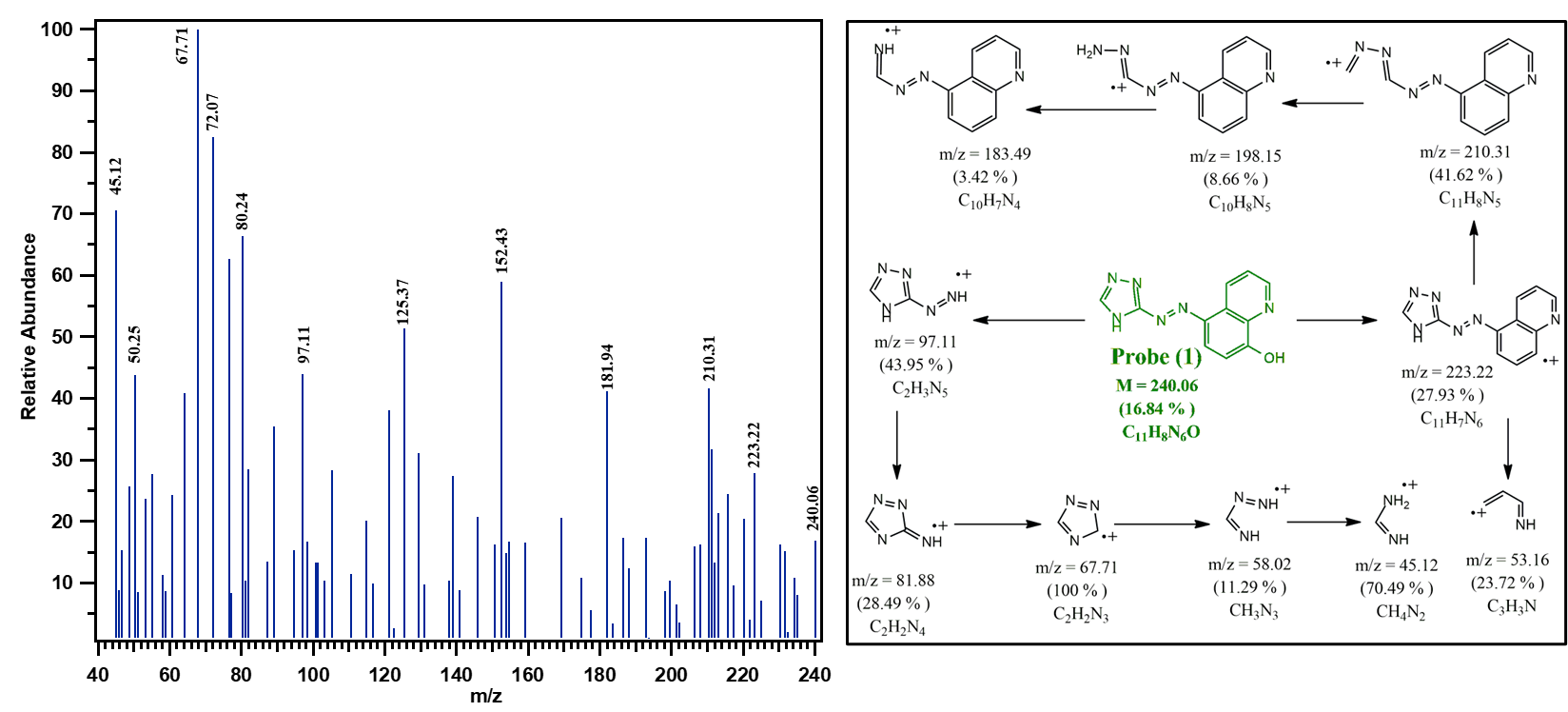


**Fig. S4**: Mass spectrum and fragmentations of probe **1**.


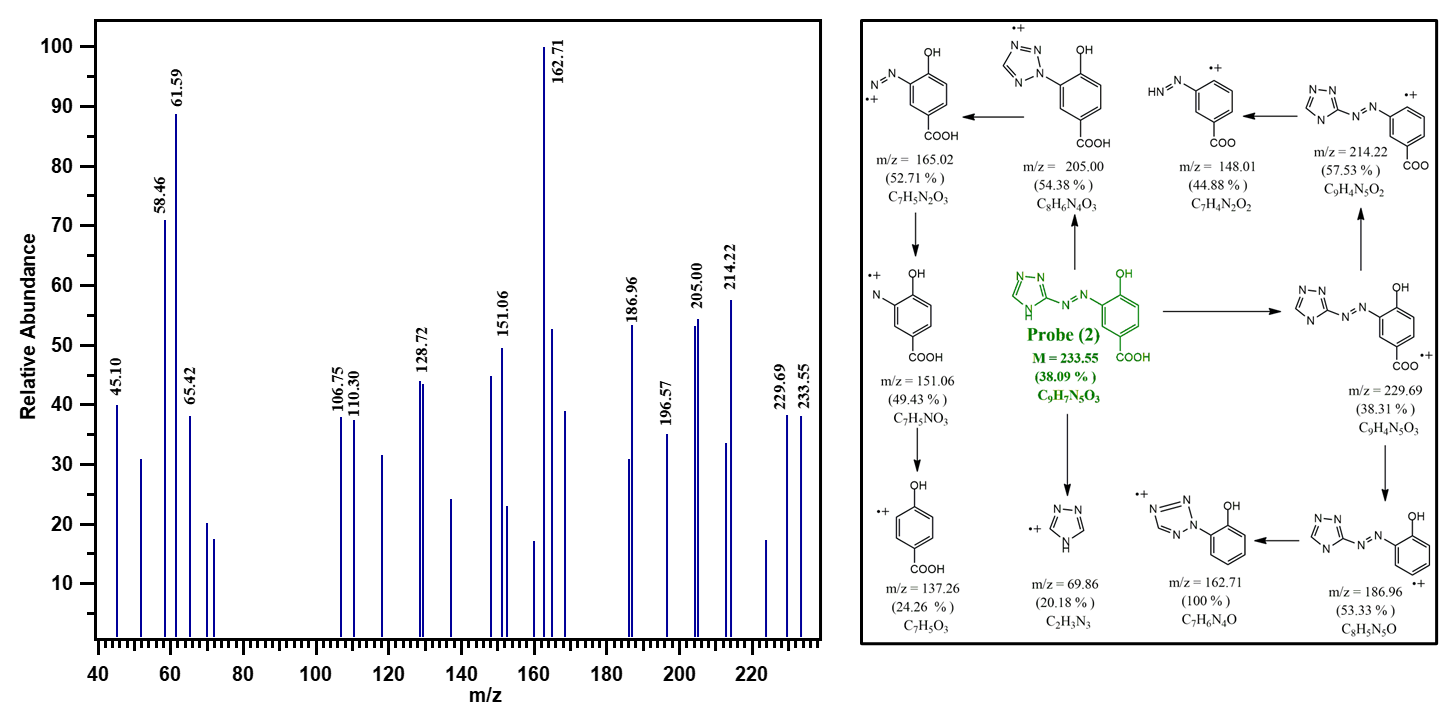


**Fig. S5**: Mass spectrum and fragmentations of probe **2**.


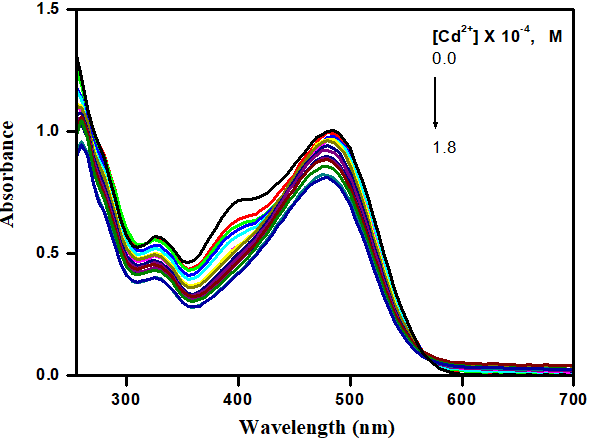

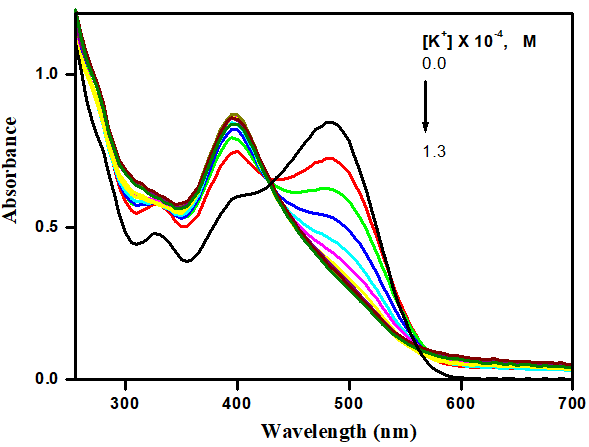

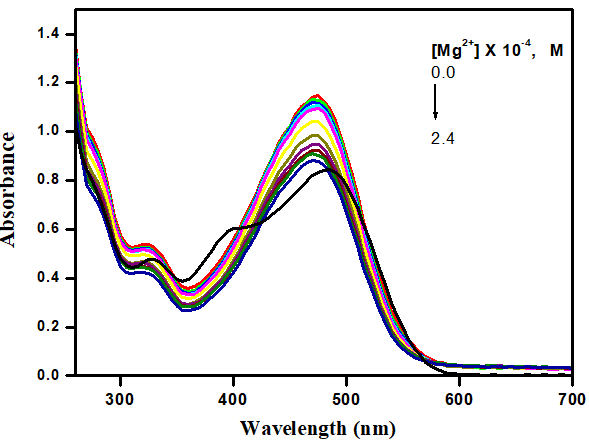

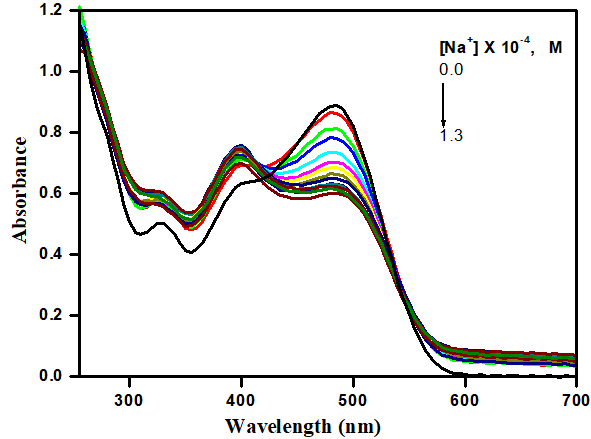


**Fig. S6**. Absorption spectral changes of fluorescent probes **(1)** upon addition of different concentrations of Na^+^, Mg^2+^, K^+^ and Cd^2+^ions in ethanolic solution.


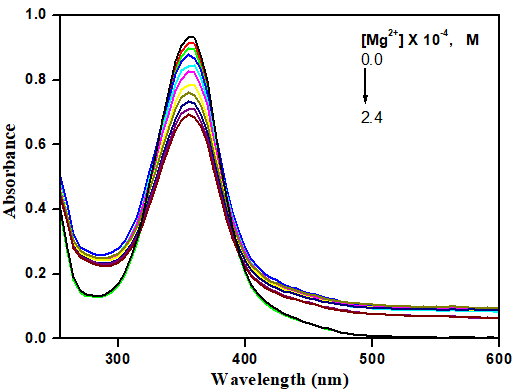

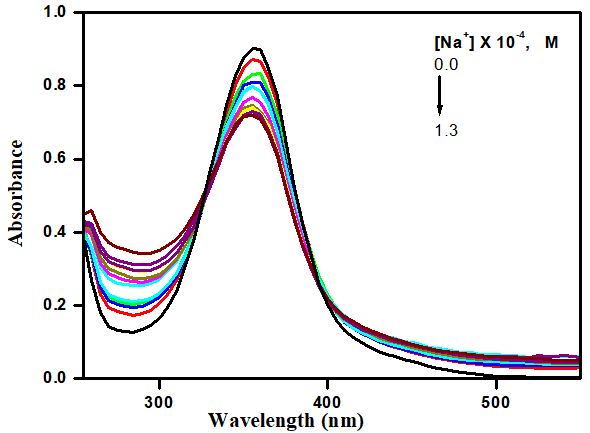


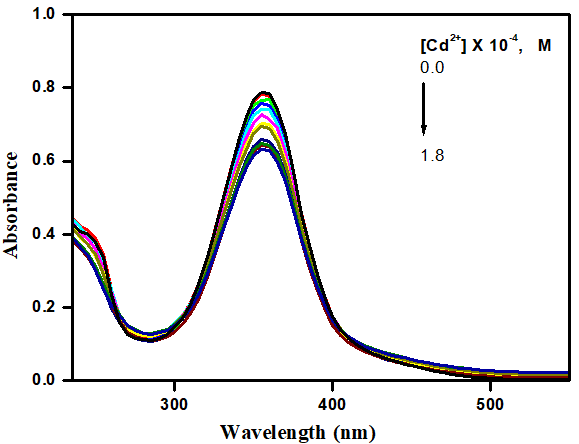

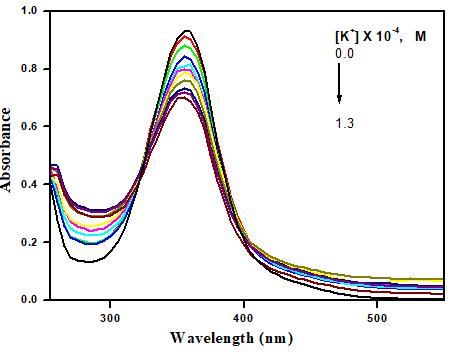


**Fig. S7**. Absorption spectral changes of fluorescent probes **(2)** upon addition of different concentrations of Na^+^, Mg^2+^, K^+^ and Cd^2+^ ions in ethanolic solution.


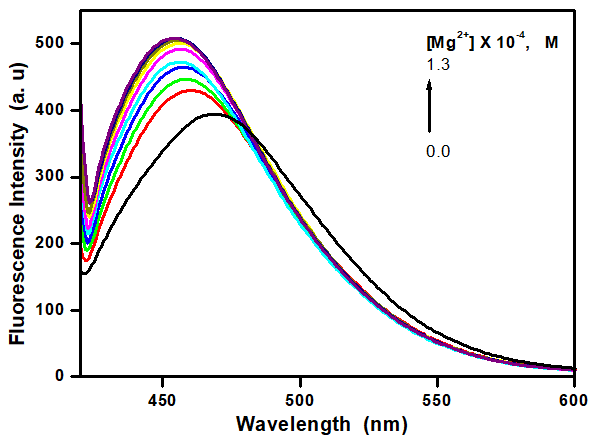

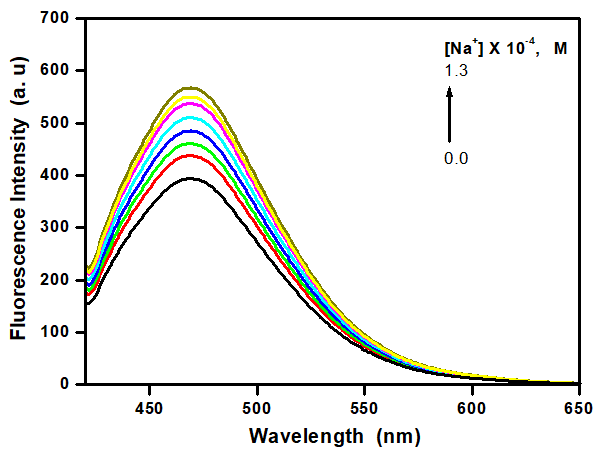


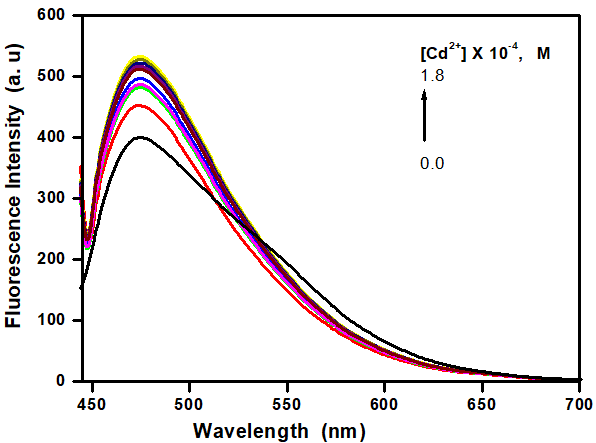

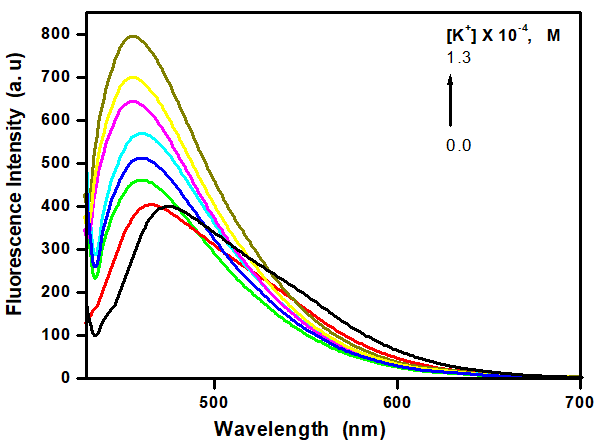


**Fig. S8**. Fluorescence spectra of fluorescent probe **(1**) upon addition of different concentrations of Na^+^, Mg^2+^, K^+^ and Cd^2+^ ions in ethanolic solution.


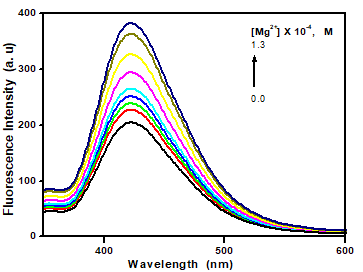

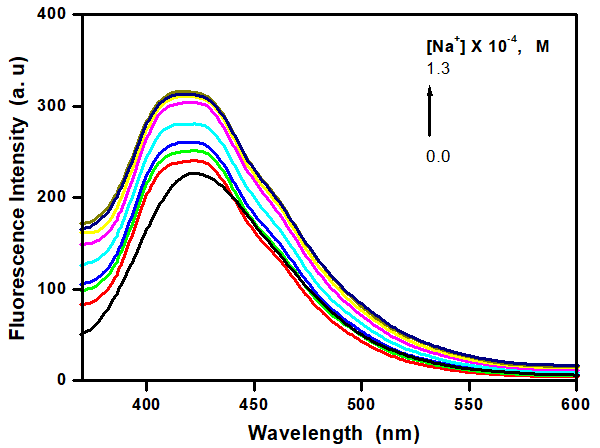


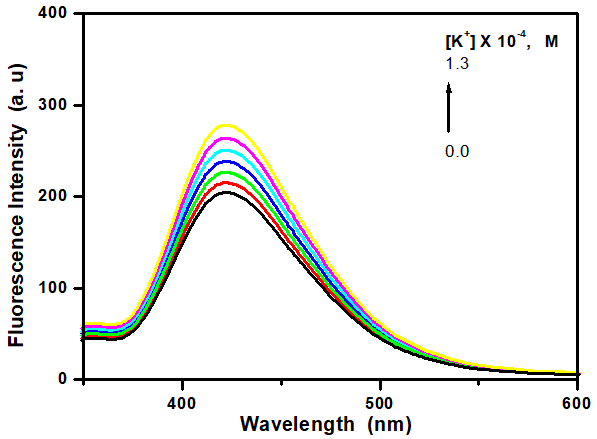


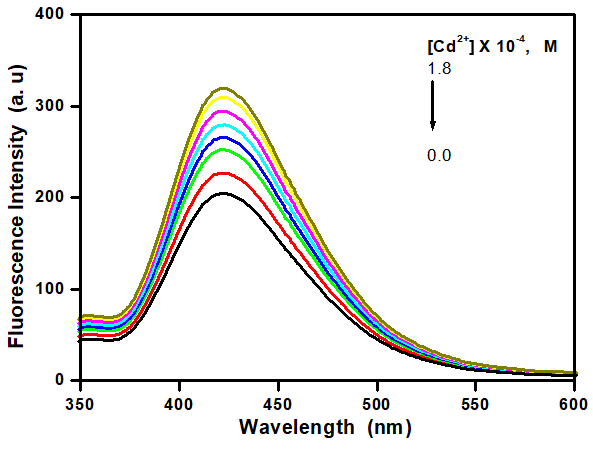


**Fig. S9**. Fluorescence spectra of fluorescent probe **(2**) upon addition of different concentrations of Na^+^, Mg^2+^, K^+^ and Cd^2+^ ions in ethanolic solution.


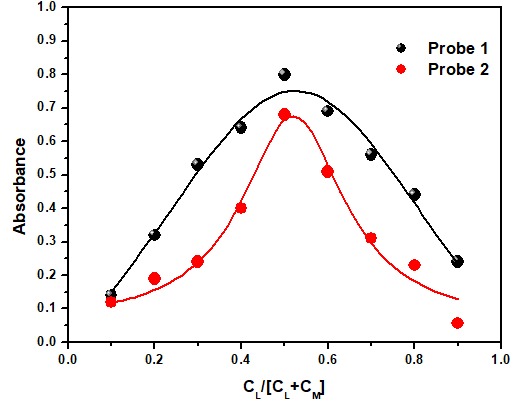


**Fig. S10**. Job's plot for the investigated probes 1 and 2 with Co^2+^ ions
